# Trafficking of immune cells across the blood-brain barrier is modulated by neurofibrillary pathology in tauopathies

**DOI:** 10.1371/journal.pone.0217216

**Published:** 2019-05-23

**Authors:** Petra Majerova, Alena Michalicova, Martin Cente, Jozef Hanes, Jozef Vegh, Agnes Kittel, Nina Kosikova, Viera Cigankova, Sandra Mihaljevic, Santosh Jadhav, Andrej Kovac

**Affiliations:** 1 Institute of Neuroimmunology, Slovak Academy of Sciences, AD Centre, Bratislava, Slovak Republic; 2 AXON Neuroscience R&D Services SE, Bratislava, Slovak Republic; 3 Institute of Experimental Medicine, Hungarian Academy of Sciences, Budapest, Hungary; 4 Department of Anatomy, Histology and Physiology, The University of Veterinary Medicine and Pharmacy, Kosice, Slovak Republic; 5 Department of Pharmacology and Toxicology, The University of Veterinary Medicine and Pharmacy, Kosice, Slovak Republic; Centre Hospitalier de l'Universite Laval, CANADA

## Abstract

Tauopathies represent a heterogeneous group of neurodegenerative disorders characterized by abnormal deposition of the hyperphosphorylated microtubule-associated protein tau. Chronic neuroinflammation in tauopathies is driven by glial cells that potentially trigger the disruption of the blood-brain barrier (BBB). Pro-inflammatory signaling molecules such as cytokines, chemokines and adhesion molecules produced by glial cells, neurons and endothelial cells, in general, cooperate to determine the integrity of BBB by influencing vascular permeability, enhancing migration of immune cells and altering transport systems. We considered the effect of tau about vascular permeability of peripheral blood cells *in vitro* and *in vivo* using primary rat BBB model and transgenic rat model expressing misfolded truncated protein tau. Immunohistochemistry, electron microscopy and transcriptomic analysis were employed to characterize the structural and functional changes in BBB manifested by neurofibrillary pathology in a transgenic model. Our results show that misfolded protein tau ultimately modifies the endothelial properties of BBB, facilitating blood-to-brain cell transmigration. Our results suggest that the increased diapedesis of peripheral cells across the BBB, in response to tau protein, could be mediated by the increased expression of endothelial signaling molecules, namely ICAM-1, VCAM-1, and selectins. We suggest that the compensation of BBB in the diseased brain represents a crucial factor in neurodegeneration of human tauopathies.

## Introduction

Neuroinflammation manifests before a significant loss of neural tissue in the process of neurodegeneration, suggesting that neuroinflammation promotes the progression of pathogenesis in neurodegenerative diseases. In neurodegenerative diseases associated with chronic neuroinflammation, immune responses driven by the main reactive components of the central nervous system (CNS) including glial cells leading to the disruption of the blood-brain barrier (BBB). Inflammatory processes affect the structure and function of BBB by increasing its vascular permeability, enhancing transmigration of peripheral blood-borne immune cells, modifying the transport systems by influencing the BBB as signaling interface [1]. Pro-inflammatory signaling molecules such as cytokines, chemokines and adhesion molecules produced by astrocytes, microglial cells, oligodendrocytes, neurons, and endothelial cells cooperate to influence the properties of BBB and regulate leukocyte-endothelial adhesion, moderate inflammation and can influence the disease pathology [2, 3]. Although the role of neuroinflammation during neurodegeneration remains unclear, findings stemming from experimental models and clinical studies have demonstrated a significant contribution of inflammation to pathological features and symptoms.

Structural and functional changes in the BBB are associated with several neurodegenerative diseases that affect CNS, including tauopathies [4]. Tauopathies are a diverse group of degenerative disorders, including Alzheimer´s disease (AD), Progressive supranuclear palsy (PSP), Pick´s disease, corticobasal degeneration (CBD), frontotemporal dementia with Parkinsonism linked to chromosome-17 (FTDP-17) and others [5, 6]. The disruption of BBB positively correlated with the progression of the pathogenesis in AD [7].

In AD, amyloid-β (Aβ) peptides are directly in contact with brain vessels [8]. A high number of patients exhibit vascular pathology and develop cerebral amyloid angiopathy (CAA) and cerebral infarcts. In patients with predominantly capillary CAA, disruption of tight junction proteins is accompanied by huge inflammatory response [9]. Recent *in vitro* studies have shown that Aβ leads to chemokine and cytokine secretion and expression of adhesion molecules that increase immune cell adhesion and transmigration of monocytes and leukocytes through the BBB [10–12]. Infusion of Aβ in rats resulted in the adhesion and migration of leukocytes across arteries, venules and cerebral vessels [13]. Significant depositions of peripheral blood-borne immune cells in the brain of AD transgenic mice was observed neighboring Aβ plaques [14]. Several evidence showed that Aβ1–42 –exposed microglia secrete TNF-α to promote trans-endothelial migration of T -cells via MHC I expression [15–17]. Alternately, studies also suggest a critical role for the infiltrating monocytes in regulating amyloid depositions in brain tissue [18].

Recent evidence showed an association between neurofibrillary pathology and progressive vascular changes that may facilitate BBB impairment in transgenic animal models and human tauopathies, including progressive supranuclear palsy, Pick´s disease [4, 19, 20] and Parkinsonism-dementia complex of Guam [21]. This implicates that tau-mediated neuroinflammation may alter BBB dynamics, which may contribute to disease progression in human tauopathies. In contrast to Aβ, very little is known about the effect of tau protein and neurofibrillary pathology on the structure and function of BBB during neurodegeneration.

Extracellular tau protein and tau oligomers play a significant role in neuroinflammatory processes [22–24]. Our previous results showed that misfolded truncated tau protein induced upregulation of CD11a, CD11b, CD18, CD4, CD45 and CD68 in the transgenic rat model of tauopathy [25]. In these transgenic animals, the expression of phagocytic markers C3, CD18, CD68, and astrocytic markers GFAP, HSP27 and VIM positively correlate with sarkosyl-insoluble tau levels in the brain areas with mature neurofibrillary pathology [26].

Tau aggregation was associated with increased production of IL-1β, IL-6, TNF-α, and MCP-1, all of which are implicated in BBB dysfunction [25, 27]. Therefore, it is likely that the tau oligomers, which form prior to intracellular neurofibrillary tangles (NFTs), and represents an early stage in tangle formation, induce inflammation and trigger changes in BBB; thus, resulting in the transmigration of peripheral blood-borne cells into brain areas affected by neurofibrillary pathology in tauopathies. In this study, we used an *in vitro* BBB model and transgenic rat model for tauopathy (line SHR-72) to investigate the role of tau protein in inducing vascular changes in the BBB, and the mechanism involved in the tau-mediated deregulation of BBB dynamics.

## Material and methods

### Animals and brain tissues

All animals used in this study were from the in-house breeding colony (SPF like, monitored according to the Federation of European Laboratory Animal Science Associations). In this study, we used transgenic rat model (line SHR-72, 6 month old) stably expressing human tau protein truncated at amino acids 151–391 (aa 151-391/4R). The transgenic rats develop progressive age-dependent neurofibrillary degeneration in the brainstem [28]. All animals were housed under standard laboratory conditions with free access to water and food and were kept under diurnal lighting conditions. All experiments on animals were carried out according to the institutional animal care guide-lines conforming to international standards (Directive 2010/63/EU) and were approved by the State Veterinary and Food Committee of Slovak Republic (RO-1101/14-221C). In survival surgery experiments each animal was stored in a separate cage and was monitored daily (food consumption, water consumption, and wound healing). Humane endpoints based on body weight loss and body condition scoring were in place. For terminal experiments, animals were euthanized with CO2. No unexplained mortality occurred in these studies. Human Alzheimer’s brain tissue was procured from Department of Psychiatry, University of Geneva School of Medicine (Geneva, Switzerland; University of Geneva brain collection; Dr. Enikö Kövari)—Female, Neuropathology: Alzheimer´s disease, Braak stage 5 [29]. All animal experiments and also experiments with human tissue were monitored by the Institutional ethics committee (Ethics Committee of Institute of Neuroimmunology)

### Expression and purification of recombinant tau protein

Human truncated tau (aa 151-391/4R) was expressed in *Escherichia coli* strain BL21(DE3) from a pET-17 expression vector and purified from bacterial lysates by size-exclusion chromatography in phosphate buffer saline (PBS; 137 mM NaCl, 2.7 mM KCl, 10 mM Na_2_HPO_4_, 2 mM KH_2_PO_4_, pH 7.4). Purified tau protein was stored under argon in working aliquots at -70°C. The purity of tau protein was verified by SDS gel electrophoresis and western blot analysis with monoclonal DC25 antibody (epitope aa 347–354 of the longest human tau isoform 2N4R; AXON Neuroscience R&D Services SE, Bratislava, Slovakia).

### Tau filament assembly and detection of tau oligomers

*In vitro* oligomerization of recombinant truncated tau protein was carried out using heparin (sodium salt from porcine intestinal mucosa, Grade I-A, cell culture tested, ≥140 USP units/mg, powder, H3149-100KU, Mw approx. 6000 g/mol, Sigma-Aldrich, St. Louis, MO) as an inducer at a final concentration of 25 μM in PBS. The reaction was performed overnight (for at least 12 hours) at 37°C. After incubation, tau oligomers were collected by centrifugation at 100 000 x g for 1 hour at room temperature, and the pellet was re-suspended in PBS and sonicated for 5 seconds at 20% power output using MS72 probe of Bandelin Sonopuls Sonifier (Bandelin, Berlin, Germany). 1 μM aliquots of aggregated protein were stored at -70°C. Oligomerization of tau protein was verified by gradient SDS electrophoresis (5–20% gel) and electron microscopy.

### Isolation of sarkosyl-insoluble tau

Sarkosyl-insoluble tau from AD brain and transgenic rat brain were isolated as previously described [30]. Briefly, 1–5 g of gray matter from human AD brain-enriched in paired helical filament tau (PHF-tau) or 200 mg of the brainstem from rat brain (n = 2) were dissected, cleaned of blood vessels and meninges and used. After sarkosyl extraction and ultracentrifugation, pellets containing PHF-tau were resuspended in 2 ml PBS and sonicated, and boiled for 5 min. Then 0.5 ml aliquots were loaded onto a stepwise 1–2.5 M discontinuous sucrose gradient and spun for 16 hours at 4°C in a Beckman ultracentrifuge using SW 32 Ti rotor (Beckman Coulter, Inc., Brea, CA, USA) at 175 000 x g. The band between 1.5 and 1.75 M sucrose interface was collected and centrifuged at 100 000 x g. The purity of the preparation was assessed by western blot analysis and electron microscopy.

### Transmission electron microscopy

Animals under tiletamine-zolazepam/xylazine anesthesia (4/2mg/kg) (CT/TG– 2/2) were perfused with PBS mixed with heparin (100 000 IU/l PBS, Sigma-Aldrich, St. Louis, MO) for 1 min and subsequently perfused with 4% glutaraldehyde (Sigma-Aldrich, St. Louis, MO) in 0.1 M cacodylate buffer (Sigma-Aldrich, St. Louis, MO). Brains were removed and fixed in the same buffer overnight. Blocks of brain tissue were post-fixed in 40 mM osmium tetroxide (Sigma-Aldrich, St. Louis, MO) in cacodylate buffer for 1 h. After rinsing in cacodylate buffer and dehydration in ethanol, samples were embedded in araldite resin. Ultrathin sections (60 nm thick) were cut using Leica EM UC6 ultramicrotome (Pragolab s.r.o, Czech Republic) and stained with uranyl acetate and lead citrate and analyzed by FEI Morgagni 268 electron microscope (Oregon, USA). For morphological examination of *in vitro* oligomerized tau and PHF-tau by electron microscopy the samples collected by ultracentrifugation were dissolved in pure water and placed on carbon-coated 400 mesh copper grids (Christine Gröpl, Austria) for 2 min. Grids were washed with pure water and negatively stained with 2% uranyl acetate for 1 min. The stained grids were immediately analyzed by FEI Morgagni 268 electron microscope.

### Isolation and cultivation of rat primary glial culture

Rat mixed glial culture was prepared from cerebral cortices of 1–2 day old Sprague Dawley rats (n = 8/isolation). The animals were euthanized by CO_2,_ and the cerebral cortices were dissected, stripped of the meninges and mechanically dissociated by repeated pipetting followed by passage through a 20 μm nylon mesh (BD Falcon, Franklin Lakes, USA). Cells were plated on 6-well plates pre-coated with poly-L-lysine (10 μg/ml, Sigma-Aldrich, St. Louis, MO) and cultivated in DMEM medium (PAA laboratories GmbH, Germany) containing 10% fetal calf serum (FCS, Thermo Fisher Scientific, Waltham, Massachusetts, USA) and 2 mM L-glutamine (Life Technologies Invitrogen, Carlsbad, CA) at 37°C, 5% CO_2_ in a water-saturated atmosphere.

### Isolation and cultivation of primary rat brain endothelial cells- *in vitro* BBB model

Isolation of primary brain endothelial cells was performed as previously described [31]. Briefly, rats (200–250 grams, 6 month old; n = 4/isolation) were euthanized by CO_2,_ and their brains were removed. Under sterile conditions, the brainstem and cerebellum were dissected, and white matter from the midbrain and the choroid plexus were removed. The cortical tissues were cleansed from meninges and were homogenized on ice in DMEM-F12 medium (PAA laboratories GmbH, Germany) with 0.1% bovine serum albumin (BSA, Sigma-Aldrich, St. Louis, MO). The homogenate was centrifuged at 800 x g for 10 min at 4°C. The supernatant was aspirated and the pellet resuspended in pre-warmed digestion mix containing 1 mg/ml collagenase/dispase (Roche Diagnostics, Indianapolis, USA) and 10 μg/ml DNase I (Roche Diagnostics, Indianapolis, USA).

The homogenates were incubated with a pre-prepared digestion mix at 37°C for 30 min with gentle shaking. The preparation was centrifuged at 800 x g for 10 min at 4°C, and the pellets were resuspended in 20% BSA in the medium. The tissues were centrifuged at 1500 x g for 15 min at 4°C to obtain pellet containing microvessels with a fraction of myelin and BSA on the top which was centrifuged again. The microvessels were pooled and re-suspended in pre-warmed digestion mix and incubated for 15 min at 37°C. The pellet was centrifuged at 800 x g for 10 min at 4°C and washed with serum containing DMEM-F12 culture medium.

The microvessels were cultured in DMEM-F12 medium (PAA laboratories GmbH, Germany) containing 15% plasma-derived serum (PDS) (First Link, UK), 2 mM L-glutamine (GE Healthcare, UK), BME vitamins (Sigma-Aldrich, St. Louis, MO), heparin (Sigma-Aldrich, St. Louis, MO) and 3 μM puromycin (Sigma-Aldrich, St. Louis, MO). After 7 days, cells were plated onto the top of 1 μm Transwell inserts (Becton Dickinson, New Jersey, USA) pre-coated with 10 μg/cm^2^ collagen type IV (Sigma-Aldrich, St. Louis, MO) and 5 μg/cm^2^ fibronectin (Sigma-Aldrich, St. Louis, MO). The cells were cultivated together with mixed glial cells plated on the bottom of 12-well plates for 7 days in EBM-2 medium (Lonza, Basel, Switzerland) containing 15% PDS, 2 mM L-glutamine, BME vitamins and BulletKit SingleQuots (Lonza, Basel, Switzerland).

### Endothelial permeability

The Transwell inserts (in a 12-well format, containing an endothelial monolayer or without cells) were transferred into 12-well plates containing 1.5 ml of Ringer-HEPES solution (150 mM NaCl, 5.2 mM KCl, 2.2 mM CaCl_2_, 0.2 mM MgCl_2_, 6 mM NaHCO_3_, 5 mM HEPES, 2.8 mM glucose; pH 7.4) per well. The cell culture medium was removed from the inserts, and 0.5 ml of Ringer-HEPES solution containing 10 μM Lucifer Yellow (LY) (Sigma-Aldrich, St. Louise, Missouri, United States) was added to the upper (luminal) compartment. All incubations were performed at 37°C. After 20, 40 and 60 min, 200 μl aliquots from each lower compartment was quantified for fluorescence intensity (Fluoroscan Ascent FL, Labsystems; excitation wavelength: 428 nm; emission wavelength: 536 nm). The endothelial permeability coefficient (Pe) of LY was calculated in cm/s^-1^. The permeability values of the inserts (PSf, for inserts with a coating only) and the inserts plus endothelium (PSt, for inserts with a coating and cells) were taken into consideration by applying the following equation: 1/PSe = 1/PSt − 1/PSf. To obtain endothelial permeability coefficient (Pe, in cm/s^-1^), the permeability value corresponding to the endothelium alone was then divided by the insert’s porous membrane surface area. The permeability value of LY in our BBB model was around 11 x 10^−6^ cm/s^-1^, below the acceptable maximum limit of 15 x 10^−6^ cm/s^-1^. The TEER values of our *in vitro* BBB model was determined by Ohm meter “EVOM” (World Precision Instrument, EVOM Sarasota, FL, USA). The TEER value of the model was approximately 300 ± 20 Ω cm^2^.

### Isolation of rat peripheral blood monocyte-derived macrophages (PB-MoM)

Rat peripheral blood monocyte-derived macrophages were obtained from peripheral blood of healthy animals anesthetized by tiletamine-zolazepam/xylazine anesthesia (4/2mg/kg) (n = 2/isolation). 4 ml of blood was diluted with sterile PBS and carefully layered on 8 ml of Ficoll-Paque Plus (GE Healthcare, UK) and centrifuged at 800 x g for 30 min at 25°C. Using sterile Pasteur pipette, the cell layer was transferred into a clean centrifuge tube and re-suspended in PBS. The cells were centrifuged at 800 x g for an additional 15 min at 25°C. Isolated cells were cultivated in RPMI 1640 medium (Thermo Fisher Scientific, Waltham, Massachusetts, USA) containing 20% FCS and 2 mM L-glutamine.

### Transendothelial migration assay of PB-MoM across *in vitro* BBB model

Rat primary endothelial cells were seeded on the upper chamber of Transwell inserts with 1 μm pore size (Becton Dickinson, New Jersey, USA) in 12-well Costar plates (Corning Incorporated, New York, USA) and grown to confluence. Three weeks old astrocytes were cultivated as an adherent monolayer and incubated with oligomeric tau (at final concentration 1μM) or PHF-tau for 48 hours. After 48 hours, the culture media were removed from glial cells and added to the bottoms of 12-well Costar plates with endothelial cells. Subsequently, 1 x 10^5^ of PB-MoM cells were loaded into the apical chamber of the inserts. After 24 hours, the PB-MoM that transmigrated into the basolateral chamber of inserts were stained with monoclonal mouse anti-rat CD45 antibody (1:300; Bio-Rad Laboratories, CA, USA) and examined by LSM 710 confocal microscope (Zeiss, Jena, Germany). In a control experiment, endothelial cells were cultivated with oligomeric tau for 48 hours. The oligomeric tau was added directly to the bottom chamber of *in vitro* BBB model. For quantification, the PB-MoM from five random fields per each insert was counted. To delineate the functional role of adhesion molecules ICAM-1 and VCAM-1 in PB-MoM binding, corresponding monoclonal antibodies (at final concentration 0.25 μg/cm^2^, all from Abcam, Cambridge, UK) were incubated with endothelial cell monolayers for 30 min prior to the addition of PB-MoM. PB-MoM that transmigrated into the basolateral chamber of inserts were stained with monoclonal mouse anti-rat CD45 antibody.

### Isolation of brain microvessels

Animals (CT/TG -12/12) were euthanized by CO_2_ overdose, and the brainstem was removed. The tissues were homogenized on ice in DMEM-F12 with 0.1% BSA. The homogenate was centrifuged at 800 x g for 10 min at 4°C. To remove myelin, the pellets were resuspended in 25 ml of BSA (20% w/v), and centrifuge at 1500 x g for 20 min at 4°C without brake. The pellets were resuspended and re-centrifuged under same conditions. The resulting pellets were resuspended in DMEM-F12 with 0.1% BSA and filtrated through 100 μm pore nylon filter (BD Falcon, Franklin Lakes, USA). The microvessels were collected by additional filtration through 20 μm nylon filters (BD Falcon, Franklin Lakes, USA). The microvessels were centrifuged at 800 x g for 10 min at 4°C. Finally, the microvessels were lysed in RLT lysis buffer and stored.

### RNA extraction and quantification of endothelial signaling molecules by quantitative real-time PCR

Rat primary endothelial cells on 6-well plates were briefly washed with 1 ml pre-warmed 1x PBS and lysed in RLT buffer (1 ml per well). Total RNA was isolated using RNeasy Mini Kit according to the manufacturer’s recommendations (Qiagen, Hilden, Germany). The genomic DNA was removed by DNase I digestion during the RNA purification. RNA was eluted into 40 μl RNase-free water. The integrity of isolated total RNA samples was determined with an Agilent 2100 Bioanalyzer using an RNA 6000 Nano Labchip kit (Agilent Technologies, Waldbronn, Germany). For transcriptomic analysis, high-quality RNA samples were used (RNA integrity number = 8.0 to 9.5). Gene expression of endothelial cells signaling molecules was performed using Rat Endothelial cell biology PCR arrays profiling 84 genes (Qiagen, Germany). Briefly, total RNA was inversely transcribed into cDNA by RT2 first strand kit (Qiagen, Germany) and 40 ng of resulting cDNA was used as a template for each qPCR reaction. Components of 25 μl qPCR reaction were as follows: 12.5 μl 2x RT2 SYBRGreen/ROX mastermix; 11.7 μl RNase-free water and 0.8 μl of cDNA (50ng/μl). Cycling conditions included an initial 95°C denaturation for 10 min, and 40 cycles of 95°C for 15 s together with amplification at 60°C for 1 min. PCR specificity was checked by melting curve analysis. Fold change of target genes was calculated using ddCt method with beta-actin (Actb) and ribosomal large protein P1 (Rplp1) as reference genes. Data in Table 1 and S1 Table represent the average of three different experiments.

**Table 1:** Inflammatory mediators associated with gene signaling in endothelial cells and brain capillaries exposed to tau proteins. Transcriptomic analysis of endothelial cells from *in vitro* BBB model and isolated capillaries from brainstem of transgenic rats (SHR72) and control animals. RT-PCR reactions were run in triplicate with Actb and Rplp1 used as the reference genes. Minimum fold change was set at ≥ 2, ≤ -2.

		**SHR72 rat model**	
**Gene symbol**	**Ref. number**	**Protein**	**Fold of regulation**
**Sele**	ENSRNOG00000002723	Selectin E	**13.9**
**Selp**	ENSRNOG00000002794	Selectin P (Granule Membrane Protein 140kDa, Antigen CD62)	**7.6**
**Cxcl1**	ENSRNOG00000002802	Chemokine (C-X-C Motif) Ligand 1	**6.3**
**Plau**	ENSRNOG00000010516	Plasminogen Activator, Urokinase	**2.8**
**Nppb**	ENSRNOG00000008141	Natriuretic Peptide B	**2.4**
**Ccl2**	ENSRNOG00000007159	Chemokine (C-C Motif) Ligand 2	**2.0**
**Mmp9**	ENSRNOG00000017539	Matrix Metallopeptidase 9	**1.9**
**Serpine1**	ENSRNOG00000001414	Serpin Peptidase Inhibitor, Member 1	**1.9**
**Tnf**	ENSRNOG00000055156	Tumor Necrosis Factor	**1.6**
**Ptgs2**	ENSRNOG00000002525	Prostaglandin-Endoperoxide Synthase 2	**1.5**
**Kdr**	ENSRNOG00000046829	Kinase Insert Domain Receptor	**-1.4**
**Mmp1**	ENSRNOG00000032353	Matrix Metallopeptidase 1	**-2.5**
		***in vitro* BBB model**	
**Gene symbol**	**Ref. number**	**Protein**	**Fold of regulation**
**Sell**	ENSRNOG00000002776	Selectin L	**361.1**
**Mmp9**	ENSRNOG00000017539	Matrix Metallopeptidase 9	**156.0**
**IL-1b**	ENSRNOG00000004649	Interleukin 1, Beta	**144.8**
**Selp**	ENSRNOG00000002794	Selectin P (Granule Membrane Protein 140 kDa, Antigen CD62)	**137.7**
**Cxcl1**	ENSRNOG00000002802	Chemokine (C-X-C Motif) Ligand 1	**107.1**
**Ccl2**	ENSRNOG00000007159	Chemokine (C-C Motif) Ligand 2	**74.7**
**Cxcl2**	ENSRNOG00000002792	Chemokine (C-X-C Motif) Ligand 2	**41.1**
**Sele**	ENSRNOG00000002723	Selectin E	**23.2**
**Vcam1**	ENSRNOG00000014333	Vascular Cell Adhesion Molecule 1	**17.5**
**IL-6**	ENSRNOG00000010278	Interleukin 6	**15.0**
**Ccl5**	ENSRNOG00000010906	Chemokine (C-C Motif) Ligand 5	**14.1**
**Ptgis**	ENSRNOG00000008245	Prostaglandin I2 (Prostacyclin) Synthase	**12.6**
**Pf4**	ENSRNOG00000028015	Platelet Factor 4	**11.9**
**Ptgs2**	ENSRNOG00000002525	Prostagladin- Endoperoxide Synthase 2	**11.8**
**Mmp1**	ENSRNOG00000032353	Matrix Metallopeptidase 1	**7.9**
**Serpine1**	ENSRNOG00000001414	Serpin Peptidase Inhibitor, Member 1	**7.8**
**Tnfsf10**	ENSRNOG00000013269	Tumor Necrosis Factor Superfamily, Member 10	**5.7**
**Icam1**	ENSRNOG00000020679	Intercellular Adhesion Molecule 1	**4.4**
**Tnf**	ENSRNOG00000055156	Tumor Necrosis Factor	**3.9**
**Cx3cl1**	ENSRNOG00000016326	Chemokine (C-X3-C Motif) Ligand 1	**3.6**
**Pdgfra**	ENSRNOG00000002244	Platelet-Derived Growth Factor Receptor, Alpha Polypeptide	**3.6**
**F3**	ENSRNOG00000011800	Coagulation Factor III	**3.4**
**Xdh**	ENSRNOG00000007081	Xanthine Dehydrogenase	**3.1**
**IL-3**	ENSRNOG00000026786	Interleukin 3 (Colony-Stimulating Factor)	**3.0**
**Ednra**	ENSRNOG00000012721	Endothelin Receptor Type A	**2.4**
**Tfpi**	ENSRNOG00000005039	Tissue Factor Pathway Inhibitor	**2.4**
**Nos3**	ENSRNOG00000009348	Nitric Oxide Synthase 3 (Endothelial Cell)	**2.3**
**Hif1a**	ENSRNOG00000008292	Hypoxia Inducible Factor 1, Alpha Subunit	**2.2**
**B2m**	ENSRNOG00000017123	Beta-2-Microglobulin	**2.2**
**Faslg**	ENSRNOG00000002978	Fas Ligand (TNF Superfamily, Member 6)	**2.1**
**IL-7**	ENSRNOG00000011973	Interleukin 7	**2.0**
**Ocln**	ENSRNOG00000018297	Occludin	**-2.1**
**Agtr1b**	ENSRNOG00000010640	Angiotensin II receptor, type 1b	**-14.2**

### Immunohistochemistry

Transgenic rats and age-matched controls (CT/TG—4/4) were deeply anesthetized with tiletamine-zolazepam/xylazine anesthesia (4/2mg/kg) and perfused intracardially with PBS. The brainstem was removed and embedded in cryostat embedding medium (Leica, Wetzlar, Germany) and frozen above the surface of liquid nitrogen. 10-μm-thick brain sections were cut on a cryomicrotome (Leica CM 1850, Leica, Wetzlar, Germany), affixed onto poly-L-lysine coated slides and left to dry at room temperature for 1 hour. Sections were fixed for 10 min in ice-cold acetone/ethanol solution and blocked for 60 min in blocking solution (DAKO, Mississauga, Ontario, Canada). Sections were incubated in primary antibodies: monoclonal mouse anti-rat CD11b (1:300; Bio-Rad Laboratories, CA, USA), monoclonal mouse anti-rat CD45 (1:300; Novus Biologicals, USA), monoclonal mouse anti-rat ICAM-1 (1:500; BD Biosciences, San Jose, CA), monoclonal mouse anti-rat VCAM-1 (1:500; BD Biosciences, San Jose, CA), polyclonal rabbit anti-rat Sele (1:250, Abcam, Cambridge, UK), polyclonal rabbit anti-rat Selp (1:250, Abcam, Cambridge, UK) monoclonal mouse anti-rat pSer202/pThr205 (AT8, 1:1000; Thermo Fisher Scientific, Massachusetts, USA), polyclonal rabbit anti-rat pThr212 (1:1000; Invitrogen Life Technologies, Carlsbad, CA), polyclonal rabbit anti-rat ZO-1 (1:250; Invitrogen Life Technologies, Carlsbad, CA), polyclonal rabbit anti-rat claudin 5 (1:250; Invitrogen Life Technologies, Carlsbad, CA), polyclonal rabbit anti-rat CD4 (1:200; Novus Biologicals, USA), polyclonal rabbit anti-rat CD3 (1:200; Novus Biologicals, USA), polyclonal rabbit anti-rat CD8 (1:200; Novus Biologicals, USA), polyclonal rabbit anti-rat pan-laminin (1: 1000, Sigma-Aldrich, St. Louis, MO). After washing, the sections were incubated 1 hour in secondary antibodies: goat anti-rabbit or goat anti-mouse AlexaFluor488/546 (1:1000; Invitrogen Life Technologies, Carlsbad, CA). Sections were mounted (Vector Laboratories, Burlingame, CA, USA) and examined using a LSM 710 confocal microscope (Zeiss, Jena, Germany).

### *In vivo* cell tracking

Animals under anesthesia induced by tiletamine-zolazepam/xylazine anesthesia (4/2mg/kg) (6 month old transgenic rats and age-matched non-transgenic controls, CT/TG– 4/4) were injected by carboxyfluorescein diacetate tracer (CFDA, Invitrogen Life Technologies, Carlsbad, CA) in 200 μl dimethyl sulfoxide (DMSO, Sigma-Aldrich, St. Louis, MO) with 10 μl heparin (Sigma-Aldrich, St. Louis, MO). Heparin was used as a precaution to avoid clotting. We injected 0.2 mg of CFDA solution per animal directly into the spleen. After 48 and 72 hours, rats were deeply anesthetized with a ketamine-xylazine cocktail and transcardially perfused with PBS. The brain was removed and embedded in cryostat embedding medium (Leica, Wetzlar, Germany) and frozen above the surface of liquid nitrogen.

### Data analysis and statistics

All experiments were repeated at least three times. Data are presented as mean ± standard deviation (SD). Statistical analysis was performed using Prism (Version 5.0, GraphPad, Inc., SanDiego, CA). Differences between means were analyzed using two-tailed Student´s t-test. For correlation, Pearson's correlation coefficient was used. Differences at p<0.05 were accepted as statistically significant. *p<0.05, **p<0.01, ***p<0.001 are used to denote statistical significance.

### Image analysis (ImageJ)

Relative staining pattern and intensity of projections from transgenic and controls rats were visualized by confocal microscopy and evaluated. ImageJ (public domain ImageJ software) was used for the evaluation and quantification of immunohistochemistry slides. We quantified 10 slices from each transgenic and control brains. For semiquantitative analysis, the color pictures were converted to grayscale 8-bit TIFF file format and regions of interest were analyzed. The grayscale 8-bit images were converted to 1-bit images, on which the number of immunolabelled structures localized in the area of interest was measured. The average intensity/pixel values of each area were then calculated, and the average intensity/pixel values representing the background intensity were subtracted from those of the immunolabelled areas. Cumulative data from every image was subjected to statistically analysis.

## Results

### Infiltration of CD3^+^ and CD4^+^ T-cells is exacerbated in the transgenic rat model for tauopathy

**Fig 1:**
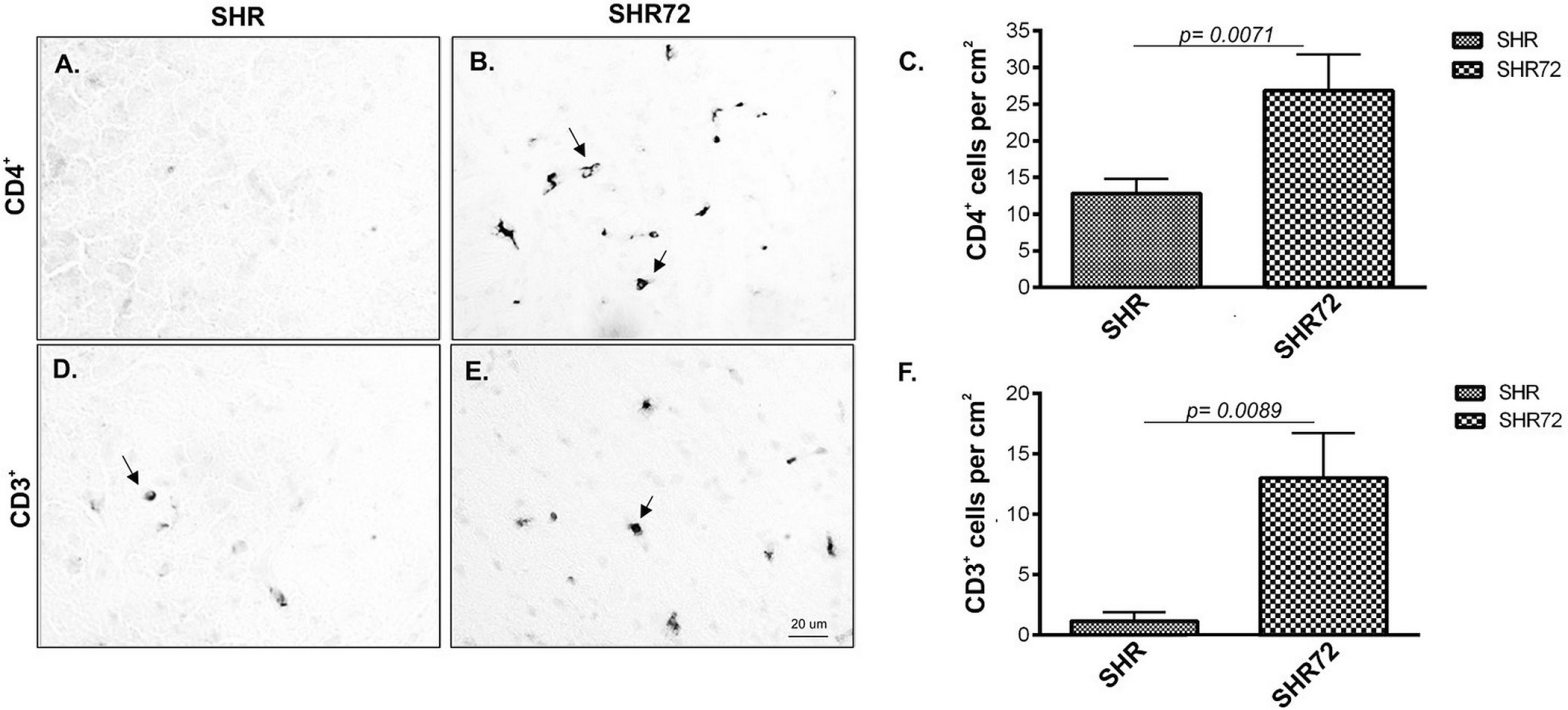
CD3^+^ T-cell and CD4^+^ T-cell occurrence in SHR-72 transgenic rat model for tauopathies compare to control animals. (A) CD4^+^ T-cell in SHR rats. (B) CD4^+^ T-cell in SHR-72 rats. (C) Quantification of CD4^+^ T-cell in control and transgenic animals. (D) CD3^+^ T-cell in SHR rats. (E) CD3^+^ T-cell in SHR-72 rats. (F) Quantification of CD3^+^ T-cell in control and transgenic animals. Scale bar 20 μm, n = 5.

We were interested in studying if neurofibrillary degeneration aggravates the infiltration of T-cells into the CNS. Therefore, we quantified the numbers of CD4^+^, CD3^+^ and CD8^+^ T-cell in the brainstem of a transgenic rodent model for tauopathy. We observed an increase in number of CD4^+^ (Fig 1A–1C; CT 12.83 ± 1.9 *vs*. TG 26.83 ± 4.96, p = 0.0071; n = 5) and CD3^+^ (Fig 1D–1F; CT 1.16 ± 0.749 *vs*. TG 13 ± 3.742, p = 0.0089; n = 5).

**Fig 2:**
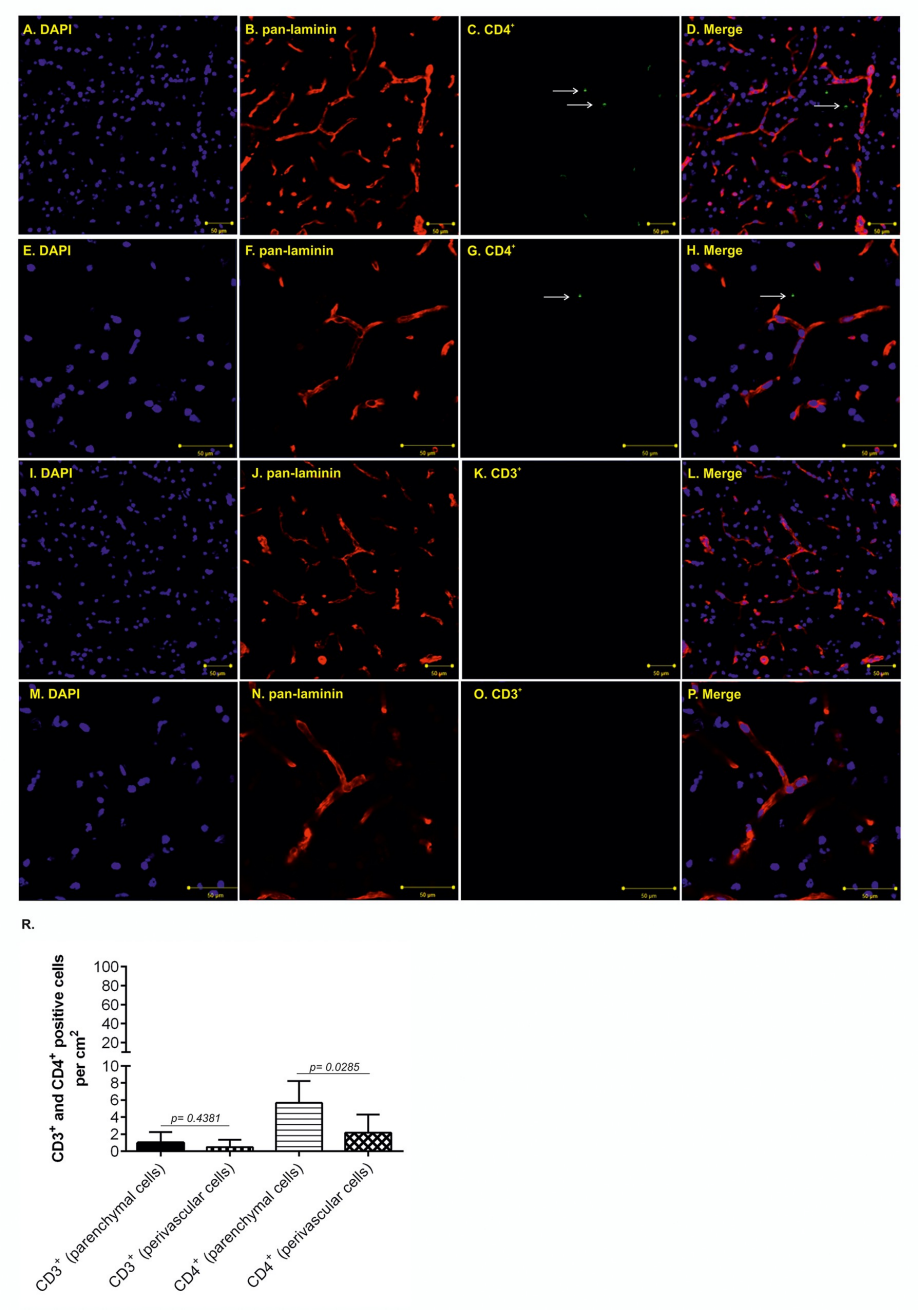
Increased CD3^+^ and CD4^+^ T-cell occurrence in the brainstem of control animals. (A-D; white arrow) Immunofluorescence staining showed more parenchymal than perivascular CD4^+^ T-cells in control animals. (E- H; white arrow) Immunofluorescence staining showed brain parenchyma infiltrating CD4^+^ T-cells in control animals, high magnification. (I-L) Immunofluorescence staining did not show the presence of parenchymal CD3^+^ T-cells in control animals. (M-P) Immunofluorescence staining did not show the presence of parenchymal CD3^+^ T-cells in control animals, high magnification. (R) Quantification of intravascular, extravascular and parenchymal CD4^+^ and CD3^+^ T-cells. Scale bar 50 μm, (DAPI: blue color; pan- laminin: red color; CD4^+^marker: green, CD3^+^ marker: green**)**, n = 7.

**Fig 3:**
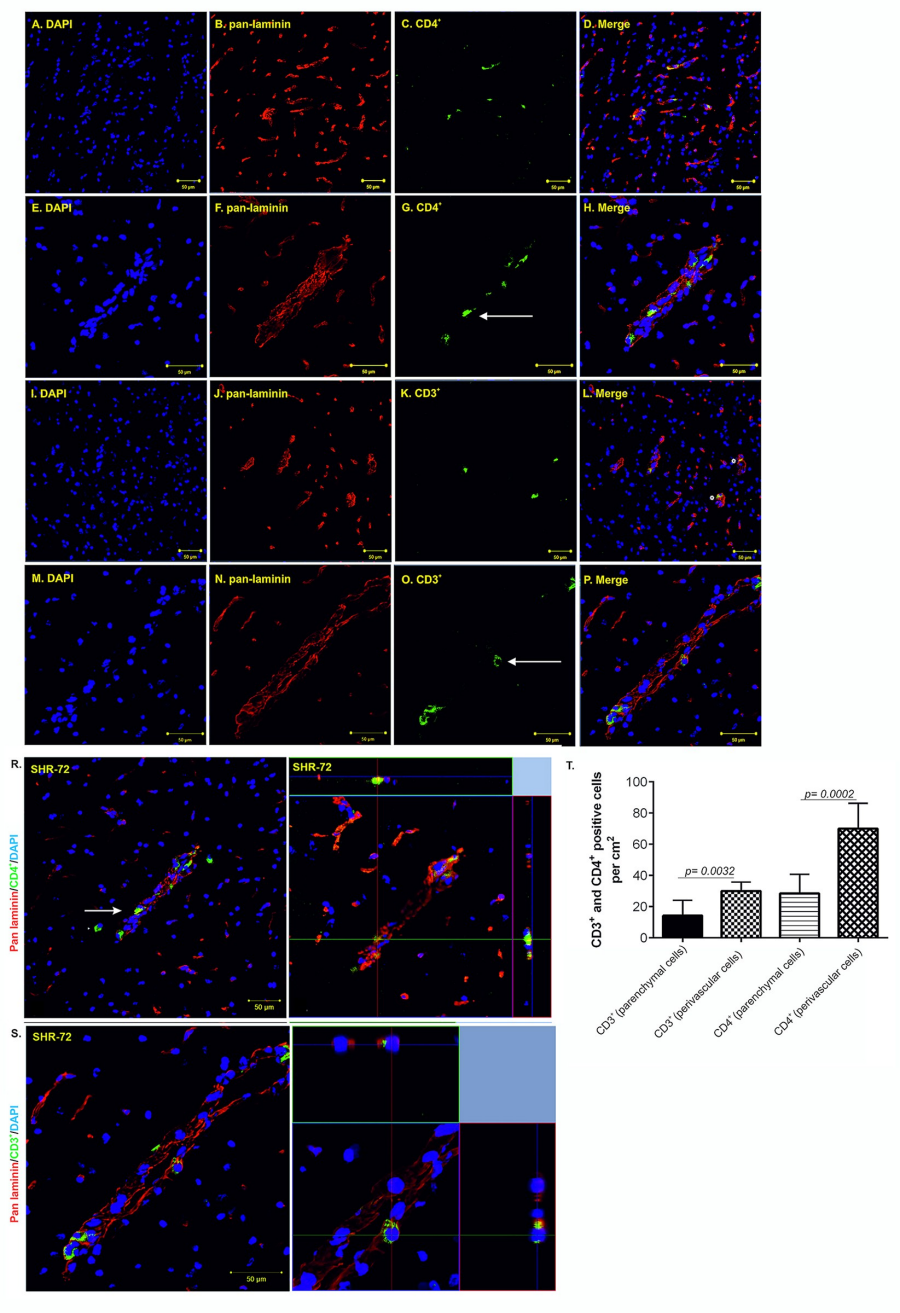
Increased CD3^+^ and CD4^+^ T-cell occurrence in the brainstem of SHR-72 transgenic rat model for tauopathies. (A-D) Immunofluorescence staining showed CD4^+^ T-cells in SHR-72 transgenic animals. (E- H) Immunofluorescence staining showed more perivascular than brain parenchyma infiltrating CD4^+^ T-cells in SHR-72 transgenic animals. (I-L) Immunofluorescence staining showed CD3^+^ T-cells in SHR-72 transgenic animals. (M-P; white arrow) Immunofluorescence staining showed mainly intravascular CD3^+^ T-cells in SHR-72 transgenic animals. (R; white arrow) Presence of CD4^+^ extravascular and parenchymal T- cells, high magnification. (S) Presence of CD3^+^ intravascular T- cells, high magnification. (T) Quantification of intravascular, extravascular and parenchymal CD4^+^ and CD3^+^ T-cells. Scale bar 50 μm, (DAPI: blue color; pan- laminin: red color; CD4^+^marker: green, CD3^+^ marker: green**)**, n = 7.

We were then intrigued if the T-cells observed in transgenic animals migrate into the brain parenchyma or are localized in the intravascular space. Using confocal microscopy, we observed retention of CD3^+^ and CD4^+^ T-cells in the perivascular space in control and transgenic rats (Figs 2 and 3). In control rats, we showed mainly the presence of parenchymal CD4^+^ T-cells (Fig 2A–2D; higher magnification Fig 2E–2H, white arrows). Compare to parenchymal cells, significantly lower number of perivascular CD4^+^ T-cells was found (Fig 2R, 5.66 ±1.05 *vs*. 2.16 ±0.87, p = 0.0285; n = 7). In contrast to CD4^+^ cells, we found only a few parenchymal CD3^+^ T-cells (Fig 2I–2L, higher magnification Fig 2M–2P). We found no CD3^+^ T-cells in intravascular space (Fig 2R, 1 ±0.51 *vs*. 0.5 ±0.34, p = 0.4381; n = 7). In transgenic animals, we showed mainly the presence of perivascular CD4^+^ T-cells (Fig 3A–3D; higher magnification Fig 3E–3H and 3R, white arrow). Significantly lower number of CD4^+^ T-cells was found in brain parenchyma (Fig 3T, 28.57 ±4.592 *vs*. 70 ±6.172, p = 0.0002; n = 7). In contrast to CD4^+^ cells, CD3^+^ T-cells accumulated in the intravascular space (Fig 3I–3L, white asterisk; higher magnification Fig 3M–3P and 3S). Lower number of CD3^+^ T-cells was found in extravascular space (Fig 3T, 30 ±2.182 *vs*. 14.29 ±3.689, p = 0.0032; n = 7). We observed only a few intraparenchymal CD3^+^ T-cells. Interestingly, the increased presence of perivascular T-cells was observed mainly the brainstem, in areas associated with neurofibrillary pathology.

### Active infiltration of PB-MoM cells into the area with neurofibrillary pathology

**Fig 4:**
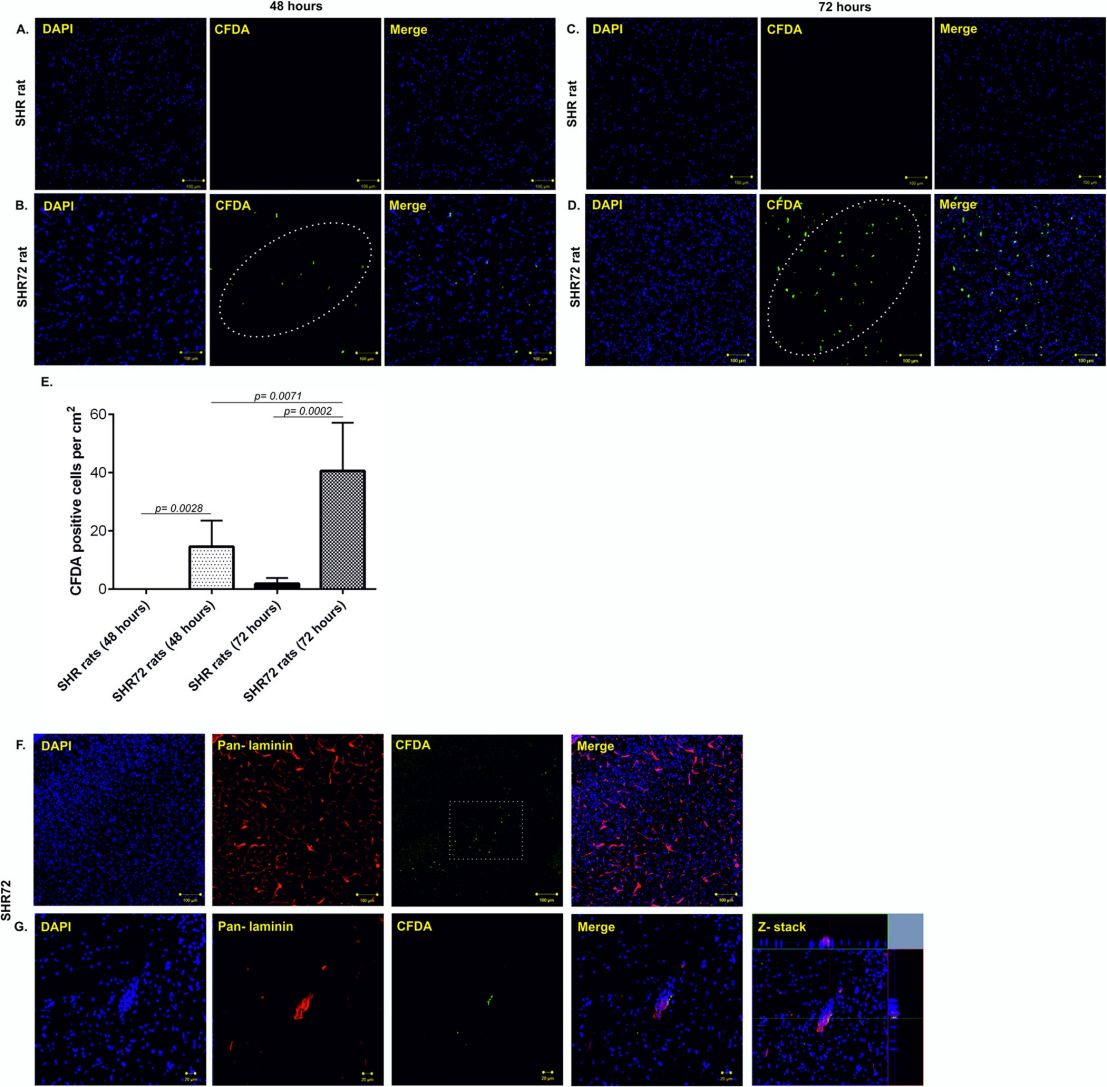
Active infiltration of CFDA—labeled PB-MoM cells into the area with neurofibrillary pathology. (A) CFDA- labeled PB-MoM cells in control SHR brain tissue (brainstem) after 48 hours. (B) CFDA- labeled PB-MoM cells in transgenic SHR-72 brain tissue (brainstem) after 48 hours. (C) CFDA- labeled PB-MoM cells in control SHR brain tissue after 72 hours. (D) CFDA- labeled PB-MoM cells in transgenic SHR-72 brain tissue after 72 hours. (E) Quantification of CFDA- labeled PB-MoM cells in the brainstem of control and transgenic rats after 48 and 72 hours. (F, G) Double immunostaining with pan-laminin showed the presence of intravascular CFDA- labeled cells. Scale bar: 100 μm and 20 μm, n = 4.

In order to confirm that peripheral cells actively migrate into the areas affected by tau neurofibrillary pathology, we used the CFDA cell labeling. After 2- and 3-days post-injection, we quantified the numbers of CFDA- labeled cells in the brain of control and transgenic animals. After 48 hours, we did not detect CFDA-labelled cells in control animals; however, we observed the presence of these cells in transgenic rat brain, mainly in the brainstem and midbrain regions (Fig 4A and 4B, CT 0 *vs*. TG 14.5 ±3.695, p = 0.0028; n = 4), the areas with neurofibrillary pathology. After 72 hours, we rarely observed CFDA- labeled cells in control tissues; however, significantly higher levels of CFDA labeled cells was observed at 72 hours in transgenic rodent brains (Fig 4C and 4D, CT 1.833 ± 0.7923 *vs*. TG 40.5 ± 6.761, p = 0.0002; n = 4). Interestingly, the number of CFDA labeled cells increased after 72 hours when compared to 48 hours (Fig 4E, p = 0.0071). Double staining with pan-laminin demonstrated that CFDA- labeled cells were found in both brain parenchyma (Fig 4F, white arrows) and perivascular space (Fig 4G).

**Fig 5:**
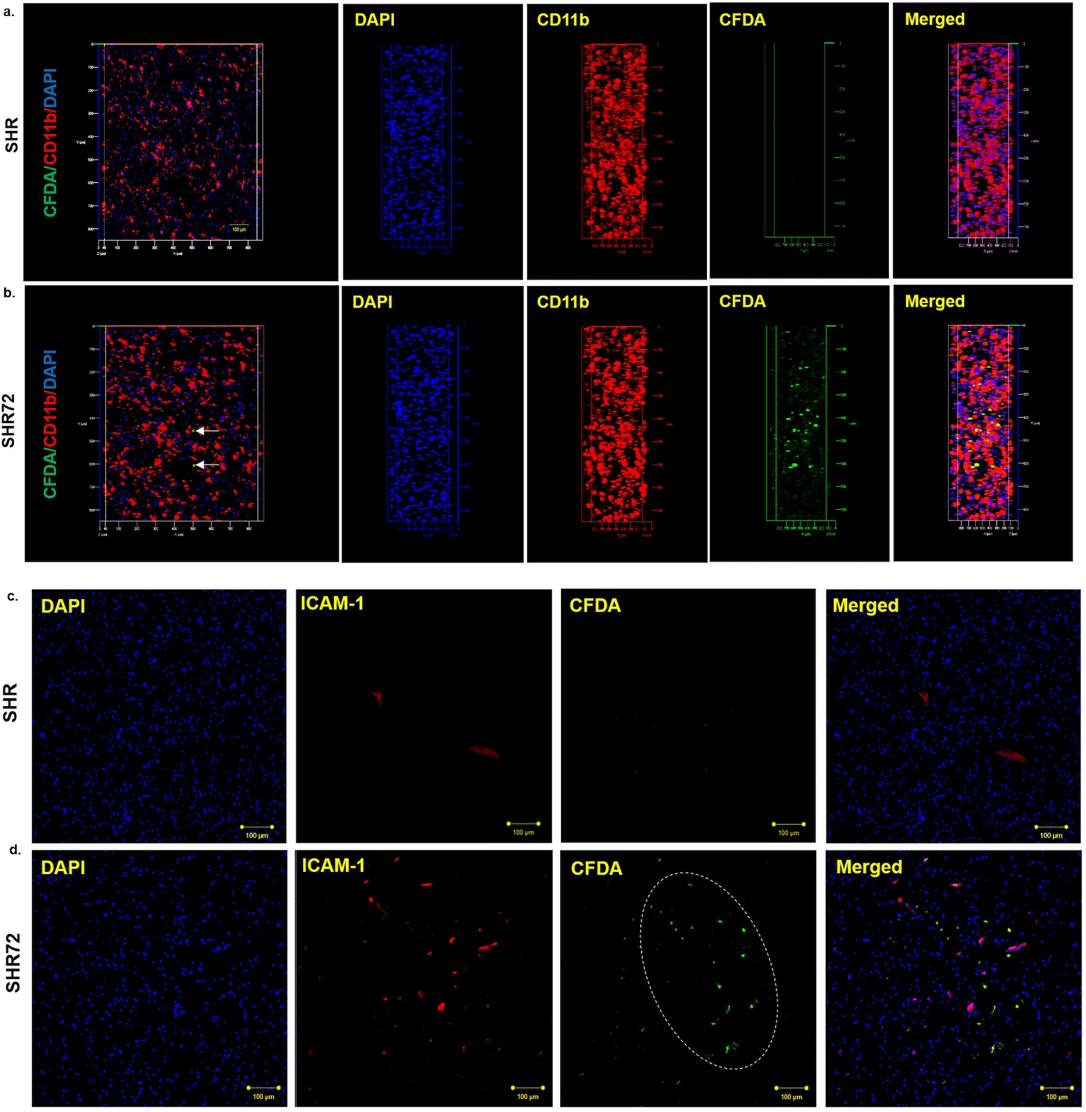
Distribution of CFDA-positive cells. CFDA-positive cells were distributed predominantly in the brainstem and middle brain of transgenic rats, areas with neurofibrillary pathology. (A, B) CFDA-positive PB-MoM cells in control and transgenic rats. In SHR72 animals CFDA-positive cells colocalized with CD11b. (C, D) Infiltration of CFDA positive PB-MoM cells in areas with increased immunostaining for ICAM-1. Scale bar: 100 μm.

Moreover, further analysis revealed that CFDA positive cells colocalized with CD11b staining (Fig 5A and 5B), confirming the infiltration of CD11b cells in the brain, from the periphery. Interestingly, the infiltration of CFDA positive cells was observed in areas with increased immunostaining for ICAM-1 (Fig 5C and 5D). This suggests that PB-MoM cells infiltrated tissue with neurofibrillary pathology associated with inflammatory changes of endothelial cells.

### Increased expression of selectins and adhesion molecules in the brain area affected by neurofibrillary tangles in a transgenic rat model of tauopathy

**Fig 6:**
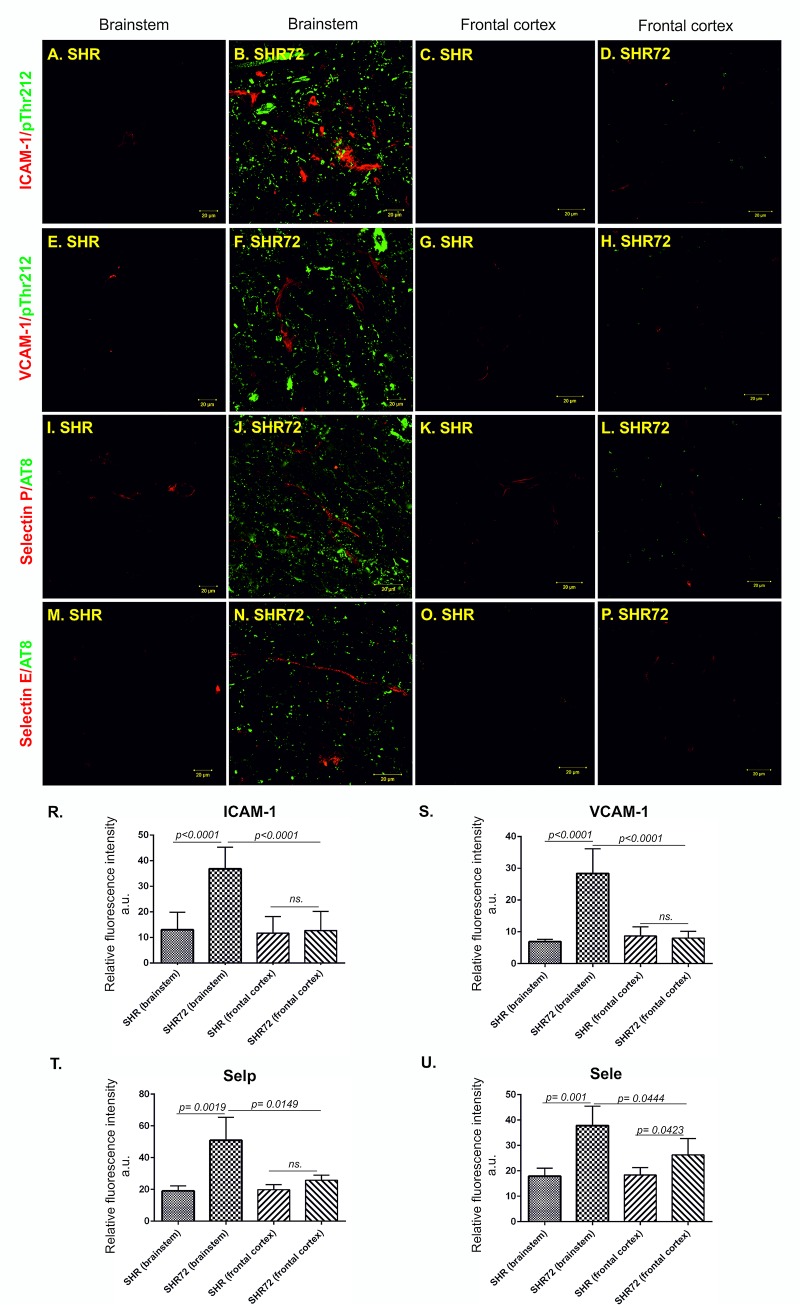
Expression of adhesion molecules is localized in the brain area affected by neurofibrillary pathology. Immunostaining of adhesion proteins ICAM-1, VCAM-1 and selectins in transgenic and control rats. Confocal microscopy showed that brain capillaries stained with ICAM-1, VCAM-1 and selectins antibodies (red color) were distributed throughout the brain affected by neurofibrillary lesions immunolabelled with pThr212 and pSer202/pThr205 (AT8, green color). Low or no signal was detected in the brainstem of control rats and frontal cortex of control and transgenic rats. (A) Presence of ICAM-1 in the brainstem of a control subject, (B) increased expression of ICAM-1 in the brainstem of transgenic rats, (C) presence of ICAM-1 in the frontal cortex of control subject, (D) expression of ICAM-1 in the frontal cortex of transgenic rats. (R) Quantification of ICAM-1 expression. (E) Presence of VCAM-1 in the brainstem of control animal, (F) in the brainstem of the transgenic animal, (G) in the frontal cortex of control animals and (H) in the frontal cortex of transgenic rats (S) with the following quantification. (I) Presence of Selp in the brainstem of controls and (J) in the brainstem of transgenic rats. (K) Presence of selp in the frontal cortex of control and (L) transgenic rats. (T) Quantification of selp immunoreactivity in control and transgenic animals. (M) Presence of Sele in the brainstem of controls and (N) transgenic rats. (O) Presence of Sele in the frontal cortex of controls and (P) transgenic animals. (U) Quantification of Sele immunoreactivity in control and transgenic animals. Scale bar 20 μm, n = 10.

We examined if the transgenic rodent model for tauopathy had deregulated expression of adhesion molecules. For this purpose, we stained the brain tissues with antibodies against intracellular adhesion molecule 1 (ICAM-1), vascular cell adhesion molecule 1 (VCAM-1) and endothelial selectins—selectin E (Sele) and selectin P (Selp), colocalized with phospho-dependent anti-tau antibodies (pThr212 and pSer202/pThr205) (Fig 6). Immunohistochemical analysis revealed significantly higher levels of ICAM-1 in the pons region of the brainstem (Fig 6A and 6B and 6R; CT 13.00 ± 2.061 *vs*. TG 36.78 ± 2.046, p < 0.0001, n = 10) of SHR-72 transgenic rats. We did not detect increased levels of ICAM-1 in the frontal cortex of transgenic animals (Fig 6C and 6D and 6R; CT 11.64 ± 1.98 *vs*. TG 12.7 ± 2.24, p = 0.727, n = 10). Increased VCAM-1 immunostaining was also observed in brainstem (Fig 6E, 6F and 6S; CT 6.897 ± 0.4123 *vs*. TG 28.35 ± 2.949, p = 0.0018, n = 10) but not in frontal cortex (Fig 6G and 6H and 6S; CT 8.674 ± 1.083 vs. TG 7.986 ± 0.8078, p = 0.6198, n = 10). Moreover, the levels of endothelial selectins Selp (Fig 6I and 6J and 6T; CT 19.00 ± 1.403 *vs*. TG 50.88 ± 7.262, p = 0.0019, n = 10) and Sele (Fig 6M and 6N and 6U; CT 17.94 ± 1.385 *vs*. TG 37.87 ± 3.777, p = 0.001, n = 10) were also elevated in the brainstem of transgenic model. However, in frontal cortex of transgenic rats we detected no significant changes of Selp levels (Fig 6K and 6L and 6T; CT 20.47 ± 1.082 *vs*. TG 24.19 ± 2, p = 0.1404, n = 10) and only slightly increased expression of Sele (Fig 6O and 6P and 6U; CT 18.38 ± 1.291 *vs*. TG 25.66 ± 2.988, p = 0.0457, n = 10).

**Fig 7:**
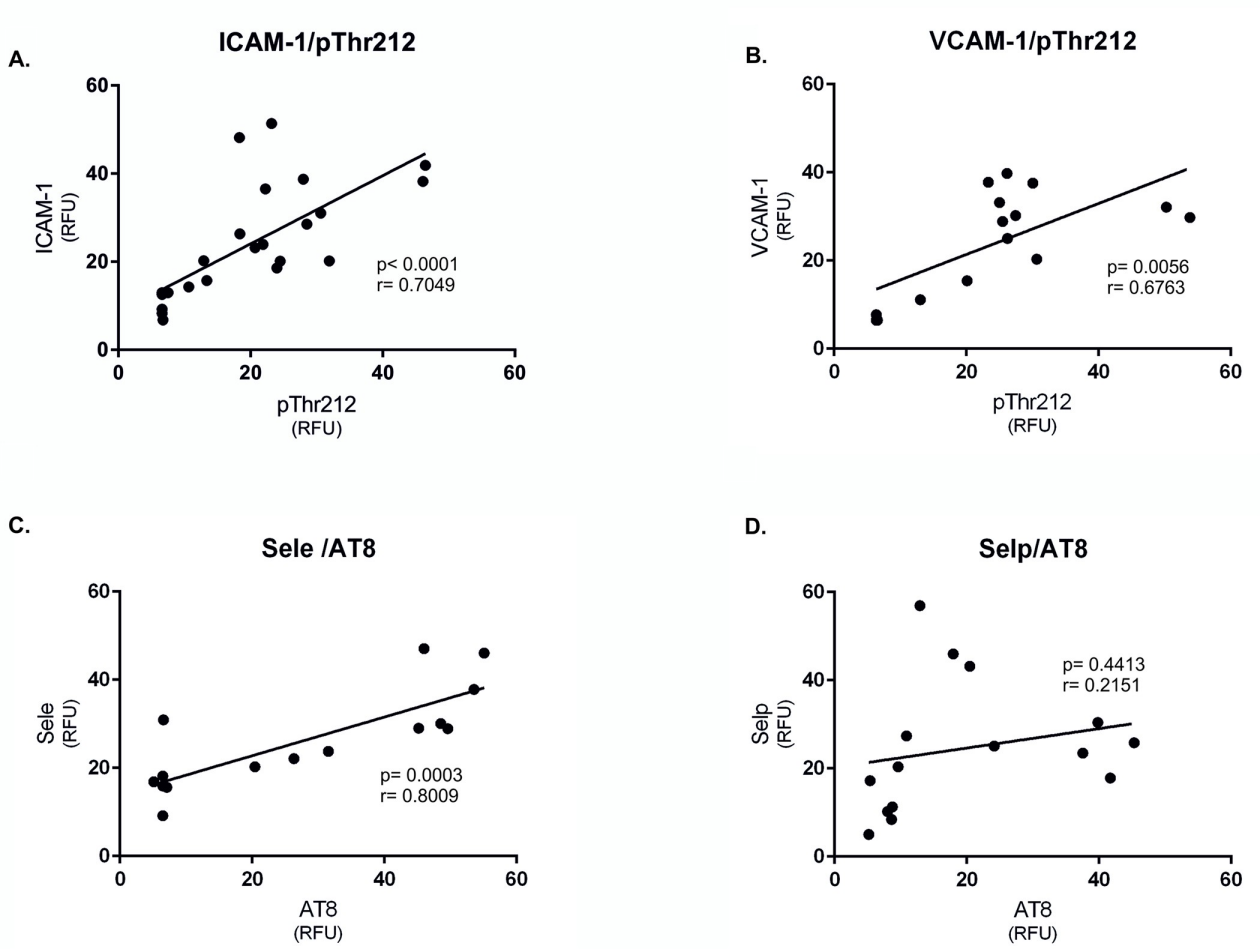
Expression of adhesions molecules correlates with the presence of neurofibrillary pathology. (A) Correlation between ICAM-1 immunoreactivity and the presence of neurofibrillary pathology (pThr212). (B) Correlation between VCAM-1 immunoreactivity and the presence of neurofibrillary pathology (pThr212). (C) Correlation between Sele immunoreactivity and neurofibrillary pathology (AT8) and (D) Selp immunoreactivity and neurofibrillary pathology (AT8).

Interestingly, the distribution of the ICAM-1, VCAM-1, and Sele strongly correlated with the presence of neurofibrillary pathology (pThr212 staining) (Fig 7A–7C); however, we did not observe any correlation between Selp and pThr212 staining in the brainstem of transgenic rats (Fig 7D).

### Basal membrane deformities are observed in the microvessels in the transgenic model of tauopathy

**Fig 8:**
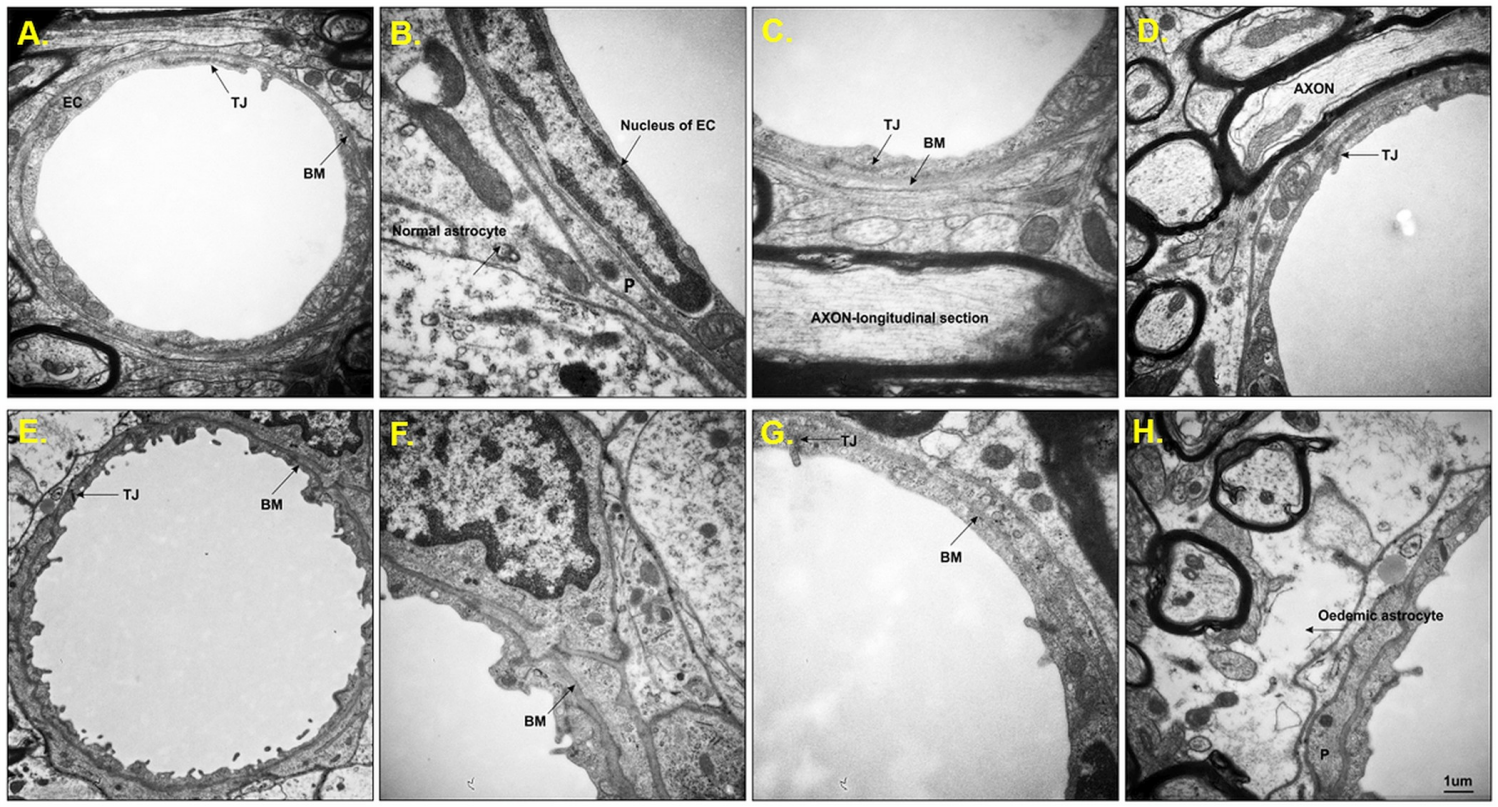
Ultrastructural analysis of brain microvessels from control and SHR-72 transgenic animals. (A) Representative brain capillaries from the brainstem of SHR control animals were characterized by normal ultrastructural appearance consisting of smooth luminal surface of endothelial cells (EC), long tight junctions (TJ) and continuous basement membrane (BM). (B) Pericyte (P) with well-defined endothelial cell nuclei surrounded by BM and normal astrocyte. (C) Represented a detailed picture of a continuous BM and (D) showed EC, closed TJ and axons. (E-H) Representative brain capillaries from the brainstem of SHR-72 transgenic animals were characterized by the wavy shape of the luminal surface with numerous processes, which clearly distinguish EC from a transgenic animal from the EC in control animals. (E) The BM is uneven (thicker/thinner), and the shape of TJ is irregular. (F) thick and uneven BM. (G) Represented a detailed picture of the discontinuity of BM and deformed TJ. (H) Electron micrograph showing oedematous astrocyte (black arrow). Scale bar 1 μm.

We performed electron microscopy to investigate if there are any structural changes in the structure of BBB in transgenic rodents. Electron imaging of brainstem from controls (Fig 8A–8D) showed normal ultrastructural appearance consisting of the smooth luminal surface of endothelial cells, surrounded by continuous and even basement membrane. Pericytes with well-defined nuclei were observed. Tight junctions (TJs) were intact, and surrounding astrocytes and myelinated axons showed normal morphology.

On the other hand, the capillaries from transgenic animals showed abnormal features (Fig 8E–8H). Numerous wavy protrusions on the luminal surface of the endothelial cells and hemidesmosome-like structures on its basal side were observed. The basal membrane was significantly uneven, and the TJs were distorted or deformed. Moreover, clear and dense oedematous astrocytes were associated with the vessels in the brainstem of the transgenic rat model.

### Misfolded tau-induced transmigration of cells across BBB is glia dependent

We were intrigued whether misfolded tau induces deregulation of the BBB and facilitate transmigration of cells across the BBB. For this purpose, we employed *in vitro* BBB model and subjected to treatment with *in vitro* prepared oligomeric tau (truncated tau aa 151-391/4R, 25 kDa), and insoluble PHF-tau isolated from transgenic rat model and human AD brain. Characterization of oligomeric tau using Western blot analysis showed the presence of high molecular weights tau species in our preparation (45–170 kDa, S1A Fig). Analysis of insoluble tau from transgenic animals showed the presence of monomeric and hyperphosphorylated truncated tau and oligomeric high molecular weight forms (S1C Fig). Moreover, the presence of endogenous tau and A68 triplet characteristic for AD were observed in PHF-tau isolated from AD brain (70–110 kDa, S1E Fig).

Morphological examination of oligomeric tau by electron microscopy (EM) revealed the presence of small circular oligomers with diameters ranging from 10 to 30 nm and short filaments up to 120 nm long with a width of 10–15 nm (S1B Fig). Also, imaging of insoluble PHF-tau from rodent and AD brain preparations showed the presence of longer filamentous and twisted structures of up to 200 nm (S1D and S1F Fig).

**Fig 9:**
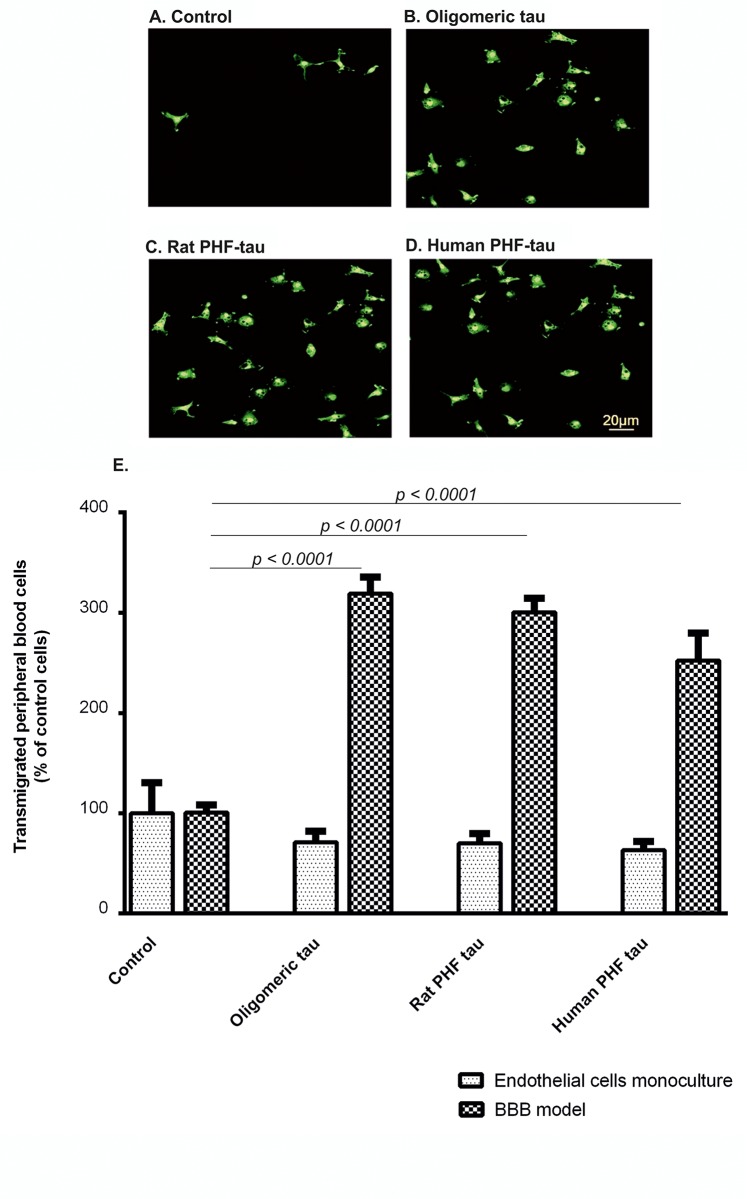
Oligomeric tau and PHF-tau stimulate PB-MoM transmigration across the *in vitro* BBB model. Effect of the interaction of oligomeric tau and PHF-tau with rat primary mixed glial cells on the transmigration of peripheral blood cells across *in vitro* BBB model. (A) After 24 hours, CD45 positive transmigrated peripheral blood cells were stained and examined by immunocytochemistry in control conditions, (B) after stimulation with oligomeric tau, (C) rat PHF-tau and (D) human PHF-tau and (E) quantified. Scale bar 20 μm. Data are means ± SD of individual experiments run in sixplicate, n = 30. Differences at p<0.05 were accepted as statistically significant.

The *in vitro* BBB model (S2 Fig) was utilized to analyze transmigration of PB-MoM across endothelial monolayer in the presence of oligomeric tau species (Fig 9A–9D). Direct abluminal exposure of brain endothelial cells alone to aggregated tau proteins did not evoke any significant responses. However, when glial cells were present, we detected increased transmigration of PB-MoM (visualized by CD45 immunostaining and counted) after 24 hours of addition of aggregated tau. In control conditions only, low amount of monocyte-input was able to transmigrate to the opposite side of Transwell inserts. In contrary, we observed accelerated transmigration of PB-MoMs with oligomeric tau by 319.0% (p < 0.0001, n = 30), rat PHF-tau by 300.6% (p < 0.0001, n = 30), and human PHF-tau to 252% (p < 0.0001, n = 30; Fig 9E) in the presence of glial cells. This suggests a scenario that interaction of oligomeric tau proteins with glial cells and their following activation augment migration of PB-MoM across the endothelial monolayer.

### Involvement of ICAM-1 and VCAM-1 in aggregated tau-mediated transendothelial migration of PB-MoM

**Fig 10:**
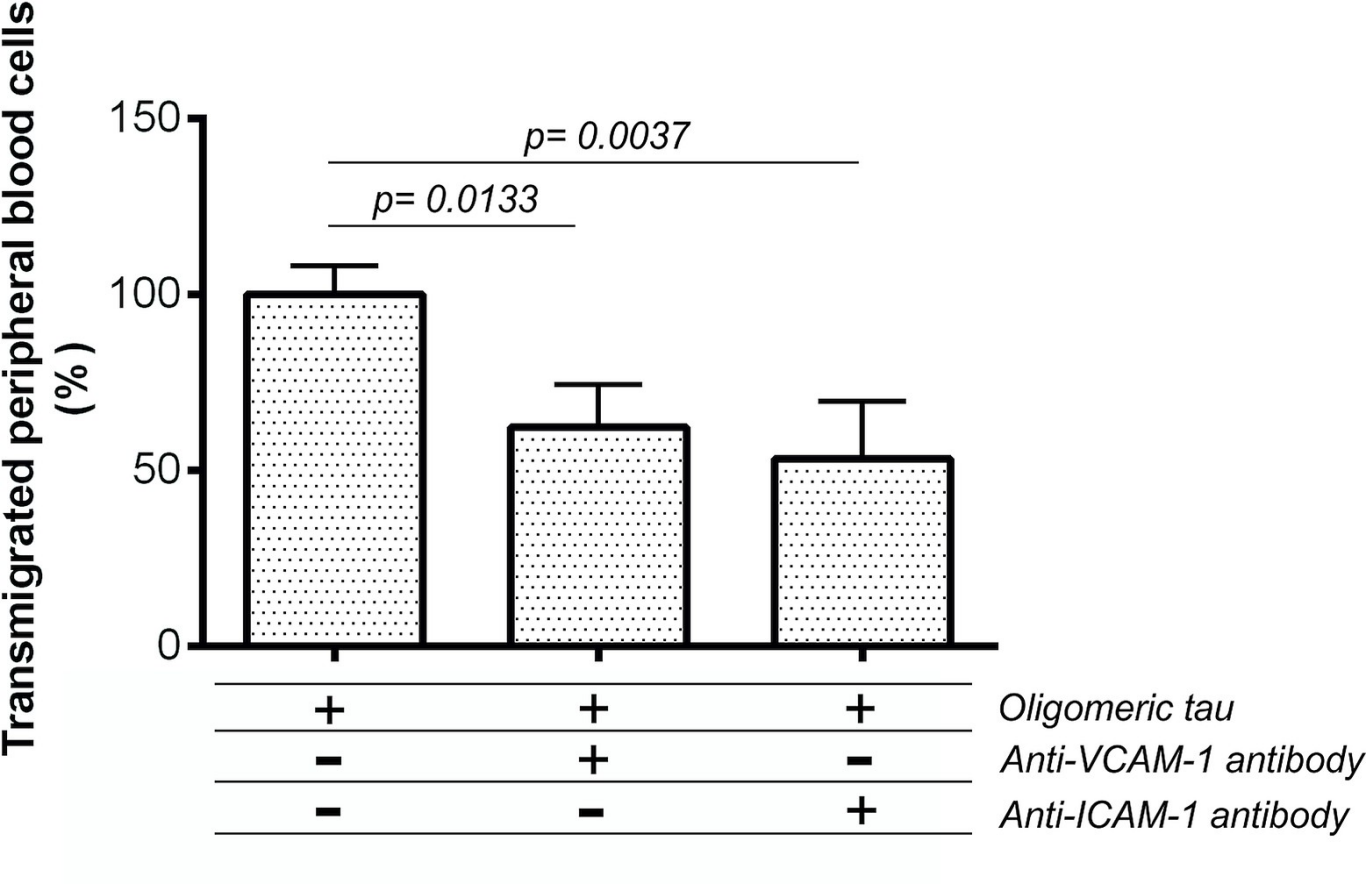
Involvement of adhesion molecules ICAM-1 and VCAM-1 in tau-mediated transendothelial migration of PB-MoM. We test the effect of antibodies ICAM-1 and VCAM-1 on transendothelial migration of PB-MoM cells across *in vitro* BBB model. The graph showed a decline in amount-percentage of transmigrated cells after antibody inhibition. Data are means ± SD of individual experiments run in six plicate, n = 30. Differences at p<0.05 were accepted as statistically significant.

We examined if the transmigration of PB-MoM across *in vitro* BBB model is mediated by adhesion molecules ICAM-1 and VCAM-1. Endothelial cells grown on Transwell inserts were preincubated with monoclonal antibodies against ICAM-1 and VCAM-1 for 30 min before the addition of PB-MoM. The pre-treatment of endothelial cells with monoclonal antibodies significantly reduced the transmigration of PB-MoM across *in vitro* BBB model (Fig 10**)**. The addition of an antibody against VCAM-1 caused a decrease in transmigration from 100% to 62% (p = 0.0187, n = 30). Monoclonal anti-ICAM-1 antibody reduced the transmigration from 100% to 53% (p = 0.0194, n = 30). These results demonstrate that the adhesion molecules play a role in regulating the flux of blood cells across the *in vitro* BBB model.

### Expression of pro-inflammatory molecules and proteins involved in diapedesis are deregulated in *vitro* endothelial cells and capillaries from transgenic tauopathy brain

We performed transcriptomic analysis of endothelial cells from *in vitro* BBB model and capillaries from brainstem of transgenic and control animals. At first, the transcriptional profile of genes that were up- or down-regulated at least 1.5fold *in vitro* conditions is shown in Table 1. Upon stimulation of *in vitro* BBB model with oligomeric tau, we found elevated expression of genes related to inflammation, leukocyte influx, endocytosis, angiogenesis, blood coagulation, and vasoconstriction. We also observed an increase in expression of genes for proteins that allow the rolling, adhesion and extravasation of PB-MoM. In capillaries from transgenic tauopathy model, upregulation for Sele, Selp, Chemokine (C-X-C Motif) Ligand 1 (Cxcl1), Plasminogen Activator (Plau), Natriuretic Peptide B (Nppb), Chemokine (C-C Motif) Ligand 2 (Ccl2), Matrix Metallopeptidase 9 (Mmp9), Serpin Peptidase Inhibitor 1 (Serpin 1), Tumor Necrosis Factor (Tnf), Prostaglandin-Endoperoxide Synthase 2 (Ptgs 2) was observed. On the other hand, the expression of Kinase Insert Domain Receptor (Kdr) and Matrix Metallopeptidase 1 (Mmp 1) was decreased. Of the all 84 genes analyzed, 8 genes were upregulated in both *in vitro* and *ex vivo* conditions: Sele, Selp, Cxcl1, Ccl2, Mmp9, Serpine 1, Tnf, Ptgs2 (S1 Table).

## Discussion

In recent years, studies investigating the dysfunction and malfunction of brain barriers in the pathogenesis of tauopathies have gained prominence. In this study, we analyzed the mechanisms by which misfolded protein tau induce structural and functional changes of BBB, and facilitate the transmigration of blood-borne cells into the brain. Using transgenic rat model of tauopathy (line SHR-72) with progressive age-dependent neurofibrillary degeneration in the brainstem [28, 32], and well established *in vitro* BBB model [31], we demonstrate that misfolded tau deregulates inflammatory, signaling and adhesion proteins in the BBB. Our results show that misfolded tau initiates signaling events leading to glial activation. Activated glial cells, in turn, aggravate transmigration of peripheral blood cells into the brain parenchyma.

According to previous studies, increased permeability of the BBB has been more related to cerebrovascular deposition of Aβ. Microvascular degeneration and various cerebrovascular abnormalities including endothelial and pericytic damage, reduced glucose transport, increased secretion of pro-inflammatory molecules as cytokines and chemokines by activated brain-resident immune cells were observed in the proximity of senile plaques [33, 34].

Very little is known about the link between the role of misfolded tau protein in instigating functional and structural impairment of the BBB. Recent studies demonstrated that tau protein is sufficient to initiate BBB impairment in tau transgenic mouse models [35, 36]. In human studies, the association between neurofibrillary pathology and progressive vascular alternations in PSP, Pick´s disease and Parkinsonism-dementia complex of Guam have been shown [4, 21]. Also accumulation of activated immune cells has been documented in animal models and the human brain, indicating a contribution of neuroinflammation to the pathogenesis of neurodegenerative disorders [37]. Expression of truncated tau protein (aa 151-391/4R) induced upregulation of immune molecules such as CD11a, CD11b, CD18, CD4, CD45, and CD68. The number of immune-reactive brain resident cells progressively increased with the NFT load, suggesting that activated glial cells are involved in the immune response targeting tau neurofibrillary pathology [25].

Additionally, chronic neuroinflammation and an immune response are driven by microglia, and astrocytes trigger the structural and functional changes of BBB [38–42]. Neuroinflammation can promote changes in brain capillaries, such as disruption of tight junction proteins, atrophy of pericytes, thickening of basement membrane due to the accumulation of basal membrane proteins. These changes can exacerbate BBB integrity, alter transport systems or influence the role of BBB as a signaling interface. Neuroinflammation can affect BBB function by increasing vascular permeability to small molecules and plasma proteins, and finally enhancing migration of immune cells from periphery into the brain parenchyma. In the present study, we show an increase in levels of CD4^+^ and CD3^+^ T-cells in transgenic animals in the brain. Moreover, we observed transmigration of CD3^+^ and CD4^+^ T-cells into the brain stem, the area with extensive neurofibrillary pathology.

Although the migration of peripheral blood cells into brain in AD remains controversial [43, 44], we were able to detect the presence of labeled CD11b^+^ cells in the brain of tau transgenic animals, in proximity to tau pathology. This suggests that peripheral blood cells infiltrate the brain parenchyma when the BBB is compensated. The invasion is likely to be an active process relying on the interaction between endothelial adhesion molecules, and their ligands expressed on the surface of cells. Two main groups of adhesion molecules are associated with the activation of microvascular endothelium. Adhesion molecules ICAM-1 and VCAM-1 belong to the immunoglobulin superfamily and endothelial selectins such as Sele and Selp.

Increased expression of adhesion molecules and selectins initiate tethering, rolling, firm adhesion and finally extravasation of leukocytes to endothelial cells surface and enhance transmigration of immune cells from periphery into the brain [2, 45]. In the transgenic model of tauopathy, we observed upregulation of both groups of endothelial adhesion molecules. Increased expression of ICAM-1, VCAM-1, Sele, and Selp was detected in the brainstem that is strongly affected by neurofibrillary pathology and invaded by T-cells. Overall, this proves the effect of tau-mediated activation of brain microvascular endothelium.

The specific mechanism of how tau affects the BBB in tauopathies remains unclear. In amyloid-burdened brain areas detrimental pro-inflammatory cytokines such as TNF-α, IFN-γ and IL-1β are likely to be the culprit. The Aβ plaques are decorated with inflammatory molecules as cytokines, chemokines, the proteins of the classical and alternative complement pathways, peroxisomal proliferators-activated receptors, proteoglycans, heat shock proteins, the metallomatrix proteinases and intercellular adhesion molecules that induced endothelial expression of adhesion molecules as ICAM-1 and VCAM-1 and enhance transmigration of immune cells from periphery into the brain [46, 47].

To characterize the mechanism/s responsible for cell transmigration in tauopathies, we performed a set of *in vitro* experiments using double BBB model utilizing primary rat brain endothelial cells and primary mixed glial culture. Tau oligomers are significantly elevated in the patient´s brain, preceded the tangle formation and had contributed to the progression of tau neurodegeneration [31, 32]. Therefore, we explored the possibility of oligomeric tau or insoluble PHF-tau derived from AD human brain and SHR-72 rat model to facilitate transmigration of peripheral blood cells. In *in vitro* BBB model, tau-induced glial activation led to release of inflammatory factors modifying endothelial properties. We observed an increase in the transmigration of peripheral blood cells across the endothelial monolayer as a result of dysfunction of endothelial cell-cell junction permeability and increased expression of inflammatory endothelial molecules. The migration of cells was blocked by an antibody to ICAM-1 and VCAM-1, indicating the role of trans-membranous proteins in the migration process. Our previous studies showed that tau proteins were not directly toxic to brain endothelial cells *in vitro*, but were able to activate glial cells [48]. We demonstrated that tau oligomers induce microglia activation and the increased expression of the inflammatory cytokines including IL-6, IL-1β, and TNF-α [25] that can promote structural and functional changes of BBB. Increased production of TNF-α and IL-1β regulates the expression of tight junction proteins: occludin-1, claudin-5, ZO-1 and ZO-2 [49, 50]. Stimulation of endothelial cells with both TNF-α and IFN-γ increases expression levels of ICAM-1 , VCAM-1 , ALCAM , MCAM , Sele and Selp and various cytokine receptors by CNS vessels [51–53]. IFN-γ promoted transendothelial migration of CD4^+^ T- cells across the BBB [54].

Transcriptomic analysis showed dysregulation of genes related to inflammation, leukocyte influx, endocytosis, angiogenesis, blood coagulation, and vasoconstriction. Similarly, in isolated brain microvessels, we found dysregulation of genes related to inflammation, leukocyte influx, endocytosis, angiogenesis, blood coagulation, and vasoconstriction. This indicates that the same pathways are activated *in vitro* and in tau transgenic animals. Changes in genes related to inflammation are consistent with previous *in vivo* studies showing that AD microvessels release significantly higher levels of several inflammatory factors than non-AD microvessels [55]. Multiple studies have shown that the BBB and inflammation both play an important role in the pathology of tauopathies. Pathologically modified forms of tau protein that are released into the extracellular environment can activate microglia [56, 57]. Another explanation for BBB changes is that neuronal tau accumulation triggers astrocytosis, causing these cells to detach from tight junctions [36]. We observed astrocyte swelling at EM, though the exact mechanism needs further investigation.

Overall, our results clearly demonstrated that misfolded truncated tau protein could activate glial cells producing a higher amount of detrimental inflammatory factors and modify endothelial properties and enhancing the migration of immune cells into the brain parenchyma. We suggest that increased diapedesis of monocytes across the BBB in response to tau protein may play a role in the pathophysiology of tau-related disorders and could contribute to an increased inflammatory burden in human tauopathies. Whether inflammatory processes modulating BBB permeability precede the process of neurodegeneration or are the consequence of disease´s pathology remains to be established.

## Conclusions

The current study highlighted the role of tau protein in BBB changes observed in tauopathies. Neuroimmune events could be crucial components of neurofibrillary degeneration in the disease´s pathology. Our data suggest that tau protein has a prominent role in regulating, perpetuating inflammation, and thus exacerbating the disease pathology.

## Supporting information

S1 Fig Characterization of tau oligomers and PHF-tau. Oligomerized tau protein (aa 151-391/4R) was prepared by *in vitro* oligomerization reaction using polyanionic inducer heparin. (A) Monomeric tau (25 kDa, apparent molecular weight on SDS-PAGE is 30 kDa) and oligomerized tau (30–170 kDa) was analyzed by western blot and visualized by monoclonal antibody DC25 (epitope 347–354 of human tau isoform Tau40, which revealed multiple SDS-stable oligomeric species. (B) Transmission electron microscopy showed tau oligomers as small round particles and short filaments (white arrows). Scale bar represents 500 nm. (C) PHF-tau isolated from transgenic rat animal was characterized by the presence of transgene and high molecular forms. (D) Electron microscopy showed the presence of long filamentous structures up to 100 nm. (E) PHF-tau isolated from AD human brain showed the presence of typical AD A68 pattern. (F) Electron microscopy showed the presence of long filamentous structures up to 200 nm.
(TIF)

S2 Fig Scheme of primary rat *in vitro* BBB model. Schematic illustration of *in vitro* BBB model and design of permeability experiments.
(TIF)

S1 Table Full list of all analyzed genes. Transcriptomic analysis of endothelial cells from *in vitro* BBB model and isolated capillaries from brainstem of transgenic rats (SHR72) and control animals. RT-PCR reactions were run in triplicate with Actb and Rplp1 used as the reference genes. Minimum fold change was set at ≥ 2, ≤ -2.
(XLSX)

## Abbreviations

Aβ: amyloid-β
AD: Alzheimer´s disease
BBB: Blood-brain-barrier
CAA: Cerebral amyloid angiopathy
CBD: Corticobasal degeneration
CNS: Central nervous system
EM: Electron microscopy
ICAM-1: Intercellular adhesion molecule 1
IFN-γ: Interferon gamma
IL-1β: Interleukin 1 beta
IL-6: Interleukin 6
NFT: Neurofibrillary tangle
PB-MoM: Peripheral blood monocyte-derived macrophages
PHF: Paired helical filament
PSP: Progressive supranuclear palsy
Sele: Selectin E
Selp: Selectin P
SHR: Spontaneously hypertensive rat
TJ: tight junction
TNF-α: Tumor necrosis factor alpha
VCAM-1: Vascular cells adhesion molecule 1
ZO-1: Zona occuldens protein 1
ZO-2: Zona occuldens protein 2
