# Supplementary figures and images for: Trafficking of immune cells across the blood-brain barrier is modulated by neurofibrillary pathology in tauopathies
Source: PLoS One. 2019 May 23;14(5):e0217216. doi: 10.1371/journal.pone.0217216 (PMC6532920; doi:10.1371/journal.pone.0217216)

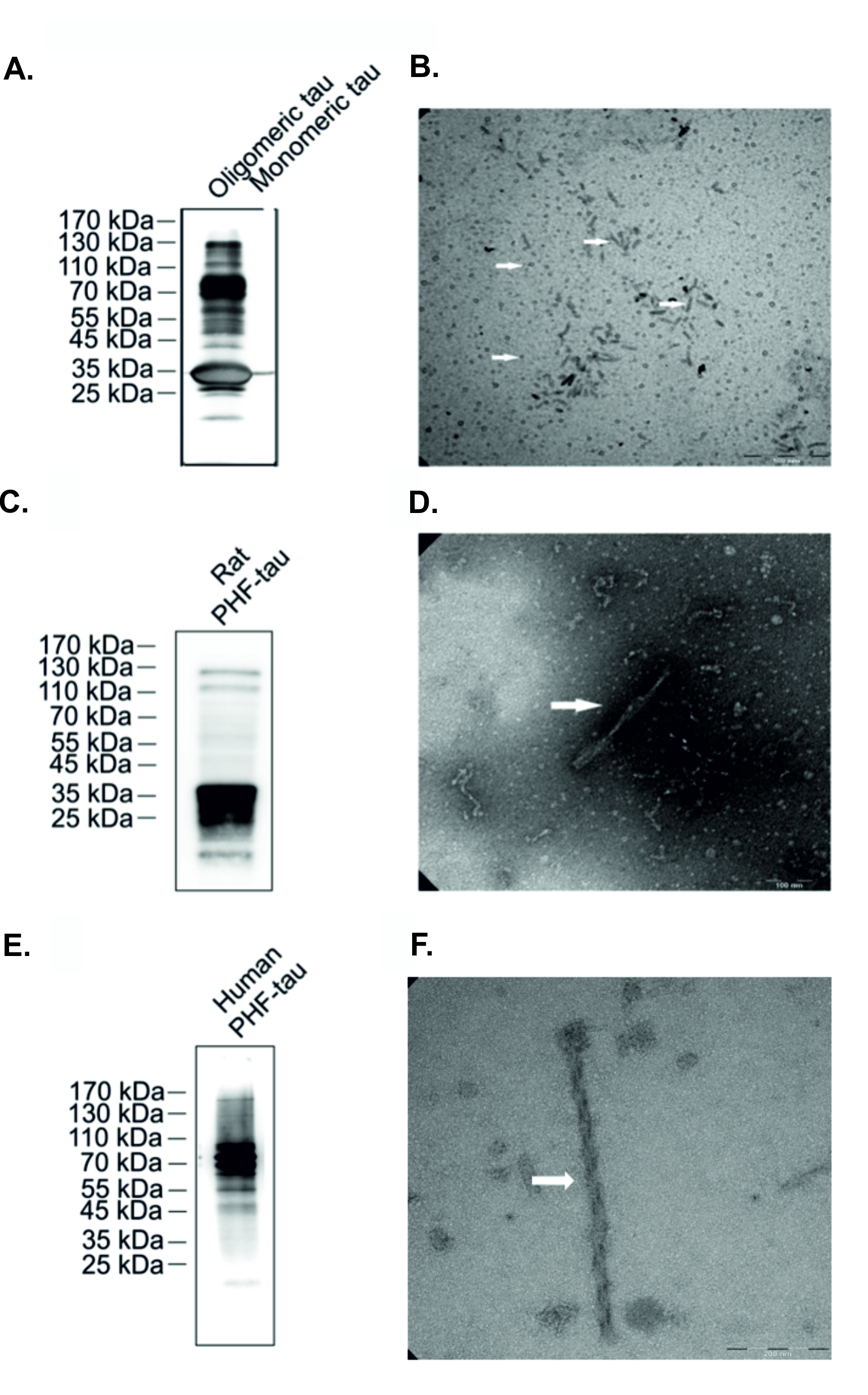

Supplement: S1 Fig — Oligomerized tau protein (aa 151-391/4R) was prepared by in vitro oligomerization reaction using polyanionic inducer heparin. (A) Monomeric tau (25 kDa, apparent molecular weight on SDS-PAGE is 30 kDa) and oligomerized tau (30–170 kDa) was analyzed by western blot and visualized by monoclonal antibody DC25 (epitope 347–354 of human tau isoform Tau40, which revealed multiple SDS-stable oligomeric species. (B) Transmission electron microscopy showed tau oligomers as small round particles and short filaments (white arrows). Scale bar represents 500 nm. (C) PHF-tau isolated from transgenic rat animal was characterized by the presence of transgene and high molecular forms. (D) Electron microscopy showed the presence of long filamentous structures up to 100 nm. (E) PHF-tau isolated from AD human brain showed the presence of typical AD A68 pattern. (F) Electron microscopy showed the presence of long filamentous structures up to 200 nm. (TIF) [file pone.0217216.s001.tif]

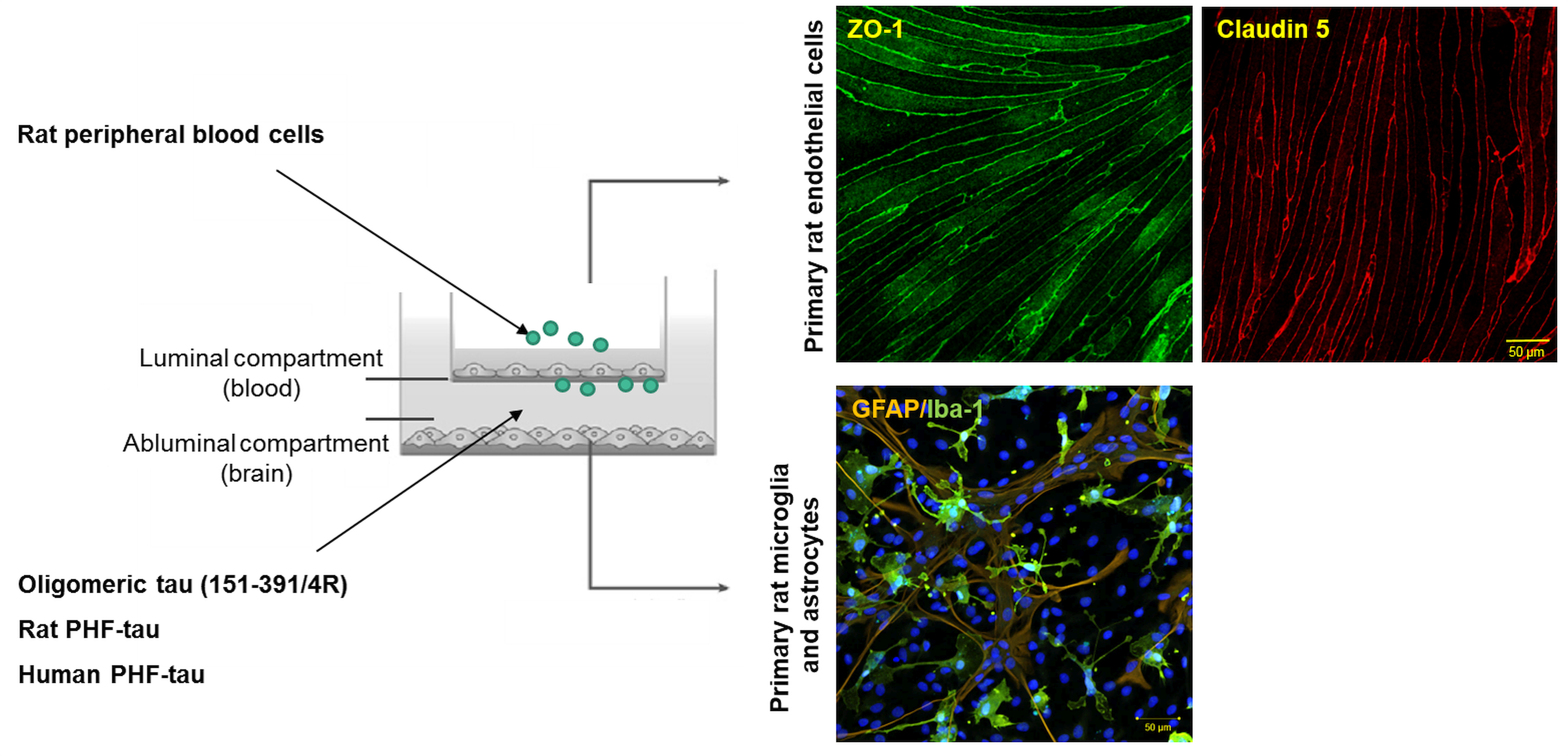

Supplement: S2 Fig — Schematic illustration of in vitro BBB model and design of permeability experiments. (TIF) [file pone.0217216.s002.tif]
